# Transcatheter mitral valve replacement for degenerated mitral valve bioprostheses, failure of mitral valvuloplasty and native valve with severe mitral annulus calcification: a systematic review and meta-analysis

**DOI:** 10.1186/s13019-021-01677-7

**Published:** 2021-10-10

**Authors:** Tao You, Wei Wang, Kang Yi, Jie Gao, Xin Zhang, Shao-E. He, Xiao-Min Xu, Yu-Hu Ma, Xin-Yao Li

**Affiliations:** 1 Department of Cardiovascular Surgery, Gansu Provincial Hospital, No. 204, Donggang West Road, Lanzhou City, 730000 Gansu Province China; 2 Gansu International Scientific and Technological Cooperation Base of Diagnosis and Treatment of Congenital Heart Disease, Lanzhou, China; 3 The First Clinical Medical College of Lanzhou University, Lanzhou, China; 4 The First School of Clinical Medical of Gansu University of Chinese Medicine, Lanzhou, China; 5 The Second Clinical Medical College of Lanzhou University, Lanzhou, China

**Keywords:** Mitral valve, TMVR, Valve-in-valve, Valve-in-ring, Valve-in-MAC, Systematic review, Meta-analysis

## Abstract

**Background:**

Although transcatheter technology has achieved some success in the field of mitral valves, the feasibility of applying it to patients with degenerated mitral valve bioprostheses (valve-in-valve, ViV), failure of mitral valvuloplasty (valve-in-ring, ViR) and serious mitral annulus calcification (vale-in-MAC, ViMAC) has not been effectively evaluated.

**Methods:**

By searching published literature before December 5, 2020 in four databases, we found all the literature related to the evaluation of feasibility assessment of TMViV, TMViR and TMViMAC. Outcomes focused on all-cause mortality within 30 days, bleeding and LVOT obstruction.

**Results:**

A total of six studies were included, and all of them were followed up for at least 30 days. After analysis of the ViV–ViR group, we obtained the following results: the all-cause mortality within 30 days of the ViV group was lower than that of the ViR group. Life-threatening or fatal bleeding was more likely to occur in the ViR group after surgery. At the same time, the ViR group was more prone to left ventricular outflow tract obstruction. However, in the ViMAC–ViR group, only the all-cause mortality within 30 days and stroke were statistically significant. In the indirect comparison, we found that TMViV had the best applicability, followed by TMViR. There were few TMViMAC available for analysis, and it requires further studies to improve the accuracy of the results.

**Conclusion:**

TMViV and TMViR had good applicability and could benefit patients who underwent repeat valve surgery. The feasibility of TMViMAC needs to be further explored and improved.

**Supplementary Information:**

The online version contains supplementary material available at 10.1186/s13019-021-01677-7.

## Introduction

Mitral valve disease is an abnormal valve structure or function caused by mucoid degeneration, congenital disease, degenerative disease and inflammation. From the latest American Heart Association (AHA) statistics, the incidence and mortality of mitral valve disease are increasing annually [1]. Patients suffering from severe mitral valve disease (insufficiency, regurgitation) were increasingly treated with annuloplasty rings or prosthetic biological valves. By analysing the data of heart valve replacement patients in California, USA, from 1996 to 2013, it was found that during this period, the utilization rate of bioprostheses during mitral valve replacement increased from 16.8 to 53.7% [2]. Due to tissue degeneration and disease progression, bioprosthetic tissue valves and natural valves that have undergone surgical repair are prone to degenerate and form lesions over time, and the vast majority of patients will require another operation [3–6]. From the current perspective, the number of repeated mitral valve operations in various heart centres around the world is increasing, and with the addition of experience, various postoperative curative effects are constantly improving. However, the risk of repeated mitral valve surgery remains higher than that of the first mitral valve surgery. Several reports have shown that the risk of repeated mitral valve surgery is very high. The 30-day mortality rate for elective mitral valve surgery was between 6.3 and 15%, and the mortality rate for emergency surgery was 17.8% [7–10]. When the third or fourth operation was required, the 30-day mortality rates for elective operations were 17.3% and 40%, respectively, while emergency operations were 40% and 44% [11]. In recent years, transcatheter mitral valve replacement (TMVR) has become an alternative to traditional cardiac surgery, and it is often used in patients with severe mitral valve disease, such as severe mitral valve bioprosthesis degradation, failure of valvuloplasty surgery, or severe mitral valve natural annulus calcification [12, 13]. Recent studies have shown that TMVR is the first choice of treatment for patients with repeated mitral valve surgery and high-risk mitral valve disease who are not suitable for traditional surgery [14].

The degenerative changes of the mitral valve bioprosthesis (valve-in-valve, ViV) and the failure of surgical rings (valve-in-ring, ViR) were largely due to the rise in life expectancy of the elderly and the short-term durability of bioprostheses compared to the mechanical mitral valve [15, 16]. After Cheung first reported transcatheter mitral valve-in-valve (TMViV) implantation in 2009 [17] and De Weger performed transcatheter mitral valve-in-ring (TMViR) replacement for the first time in 2011 [18], an increasing number of patients received these two types of surgery and benefited from them. Compared with traditional surgery, patients have achieved some efficacy after using TMVR. Nevertheless, the ultimate results remain unsatisfactory due to the patients' relatively poor baseline characteristics and various comorbidities, especially patients who received transcatheter mitral valve-in-mitral annulus calcification (TMViMAC) [15]. At one time, some clinicians doubted the feasibility of TMVR. The earliest experience of TMVR with severe mitral annulus calcification (MAC) was collected in the TMVR of the MAC Global Registry, reporting a mortality rate of 25% at 30 days [19]. A follow-up study from the multicentre TMVR registry reported a 30-day mortality rate of 34.5% [20]. In the existing reports, we found that the relatively high mortality rate was due to severe comorbidities and technical challenges related to calcium load [20, 21]. Although the use of transcatheter mitral valve replacement for patients with severe mitral valve ring calcification still had a high mortality rate, it must be admitted that compared with traditional mitral valve surgery, TMVR has become an urgent and preferred treatment for high-risk severe mitral valve disease.

With the development of the catheter era, it is necessary to evaluate the feasibility, pros and cons of TMVR for degenerated mitral valve bioprostheses, mitral valvuloplasty failure and serious mitral annulus calcification. From the first report to the present [22], many studies have been published, and there are also some authoritative statistical results from multiple centres. However, to the best of our knowledge, there have been no systematic reviews assessing the early postoperative mortality and complications of TMVR for degenerated mitral bioprostheses (ViVs), failed surgical rings (ViRs), and native valves with severe mitral annular calcification (ViMAC). In this article, we performed a meta-analysis and systematic review on the results of ViV, ViR and ViMAC to provide a reference for the selection of operation methods for patients with indications.

## Material and methods

### Protocol and registration

This systematic review and meta-analysis was performed according to the preferred reporting items for systematic reviews and meta-analyses (PRISMA) statement. The protocol was registered on INPLASY (202130113) and is available in full on inplasy.com (https://inplasy.com/inplasy-2021–3-0113).

### Publication selection

The search terms were determined through the "PICO" principle, systematic electronic searches were conducted in PubMed, Embase, Web of Science and the Cochrane Library, and the references of the included documents were manually searched to identify other publications. The time was from the establishment of the database to December 5, 2020. The purpose was to find all relevant documents on transcatheter mitral ViV, ViR and ViMAC.

Search terms were Valve-in-Ring, Valve in Ring, ViR, Valve-in-Valve, Valve in Valve, ViV, Valve-in-Mitral Annular Calcification, Valve in Mitral Annular Calcification, ViMAC.

The retrieval strategy is shown below using the Web of Science as an example:

**Table:** 

# 1 TS = (Valve-in-Ring) OR TS = ("Valve in Ring") OR TS = (ViR)
# 2 TS = (Valve-in-Valve) OR TS = ("Valve in Valve") OR TS = (ViV)
# 3 TS = (Valve-in-Mitral Annular Calcification) OR
TS = ("Valve in Mitral Annular Calcification") OR TS = (ViMAC)
# 4 #2 AND #1
# 5 #3 AND #1
# 6 #3 AND #2
# 7 #4 OR #5 OR #6

### Inclusion and exclusion criteria

#### Inclusion criteria

1. Articles written in English. 2. Minimum of 30 days follow up post-procedure. 3. The subject of the study was the outcomes of TMVR for patients with degenerated bioprostheses [valve-in-valve (ViV)], failed annuloplasty rings [valve-in-ring (ViR)], and severe mitral annular calcification [valve-in-mitral annular calcification (ViMAC)]. 4. The research included ≥ 10 patients undergoing either ViV–ViR, ViR–ViMAC or ViV–ViR–ViMAC.

#### Exclusion criteria

1. Meeting abstracts, comments, case reports, letters and expert opinions. 2. Duplicate publication of data or unable to extract data. 3. Except for mitral annular calcification, TMVR represents the native mitral valve. 4. The study lacks main details about postprocedure results. 5. Animal-based studies.

### Data extraction and quality assessment

When we determined the final inclusion of the literature, we carefully read the full text and extracted the following data: general information of the literature (first author, publication time and country); baseline characteristics such as age, female sex, diabetes mellitus, hypertension, atrial fibrillation and 14 other pieces of information; all-cause mortality, bleeding and the other nine major recent outcome indicators (within 30 days).

All the included literature was evaluated from three aspects through the Newcastle–Ottawa Scale (NOS) scoring standard: population selection, comparability and outcome. There were nine questions in total, and the highest score was 9 points. It was generally believed that when the score was ≥ 7, the study was considered high quality [23]. Among the scoring items, except for the fifth scoring standard, which could be up to two points, the other items were all one point [24].

This part was independently conducted and cross-checked by two researchers and discussed and resolved in case of differences.

### Statistical methods and data processing

All analyses were conducted using RevMan 5.4 (http://ims.cochrane.org/revman) [Computer program]. We chose unadjusted raw data because various studies have not adjusted for the same set of confounding factors. Categorical variables are expressed as the number of occurrences, and the effect measure was the odds ratio (OR). Continuous variables are expressed as the mean ± SD. When the unit of measurement was consistent, the mean difference (MD) was used; otherwise, the mean difference (SMD) was used. A standard confidence interval of 95% (95% CI) was applied in all analyses. The Q and *I*^*2*^ tests were used for statistical heterogeneity analysis. When *I*^*2*^ > 50% or *P* < 0.1, the random effects model was adopted; if not, the fixed effects model was adopted. The test level α = 0.05, which means that when the *P* value is < 0.05, it is considered statistically significant.

## Results

### Literature selection and study characteristics

**Fig. 1:**
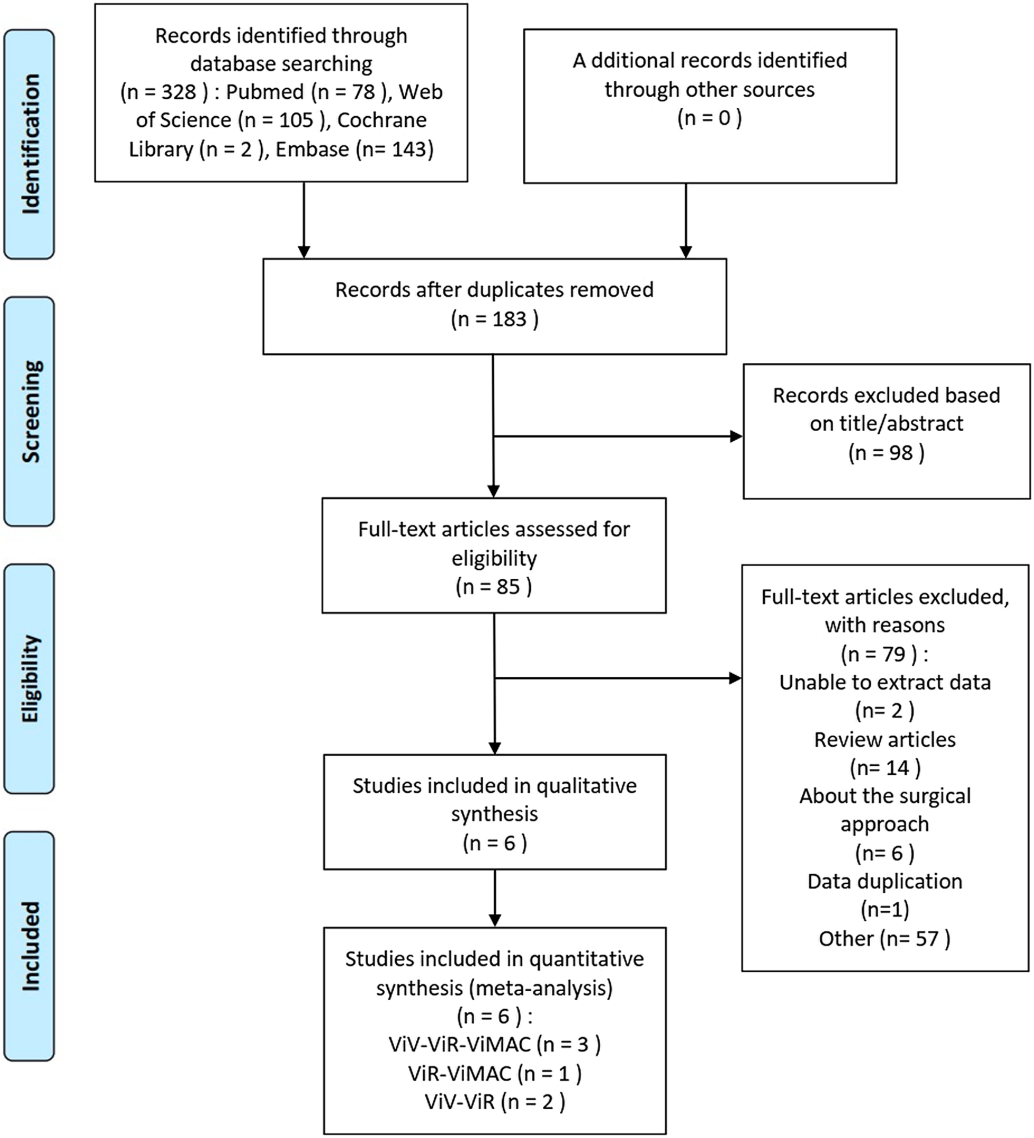
The flow chart of the literature search for this meta-analysis

**Fig. 2:**
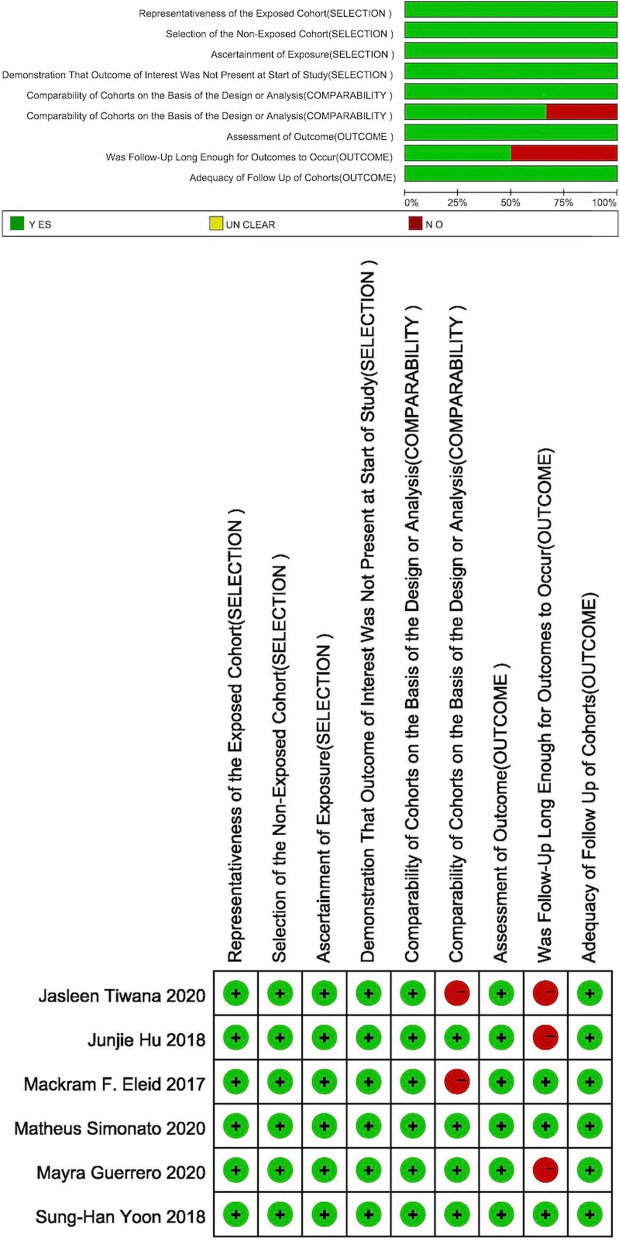
The NOS score result of the included literature output by Revman

According to the search strategy and inclusion and exclusion criteria, a total of six documents were included in this meta-analysis (see Fig. 1). Two studies researched ViV and ViR patients, one study included ViR and ViMAC patients, and three studies simultaneously researched ViV, ViR and ViMAC patients. In these studies, Mackram [25], Yoon [20], Jasleen [27] and Matheus [28] all achieved a complete follow-up of 30 days. Additionally, Yoon [20] and Matheus [28] also analysed the possible influencing factors of all-cause mortality. These factors were of great significance for evaluating the postoperative efficacy of TMVR in the absence of a randomized controlled trial in the field. Tables 1 and 2 shows detailed baseline characteristics as well as the number of deaths in each group and number of major adverse events. After reading the full text carefully, we used the NOS scoring standard to score the included literature. For the question of COMPARABILITY, we gave the literature two points when the researchers analysed other problems in addition to the main complications. Regarding the second question in OUTCOME, we believed that if the follow-up time was within 6 months, it was not long enough, and no points would be given. In the end, the highest score of our included literature was 9 points, the lowest was 7 points, and the overall quality was high. The results are shown in Fig. 2.

**Table 1:** Part of the baseline characteristics of the population included in each group

Author	Nation	Year	Consecutive cases	Follow-up	Complete follow-up for 30-day	All patients			Age (years)			Female		
						ViV	ViR	ViMAC	ViV	ViR	ViMAC	ViV	ViR	ViMAC
Eleid [25]	US	2017	Yes	30-day and 1-year	Yes	60	15	12	75 ± 11	72 ± 8	79 ± 9	34	9	5
Yoon [20]	US	2018	Yes	1, 6, and 12 months and yearly thereafter	Yes	322	141	58	72.6 ± 12.9	71.7 ± 9.7	74.7 ± 10.8	189	52	41
Hu [26]	–	2018	NO	1and 6 months	No	172	73		74.5 ± 12.5	70.0 ± 10.8		61	21	
Tiwana [27]	US	2020	Yes	30-day	Yes		12	28		74.1(71.1–77.4)	74.2(70–81.7)		8	22
Guerrero [28]	US	2020	unclear	30-day	No	680	123	100	76 (67–83)	73 (67–79)	77 (65–82.5)	407	59	69
Simonato [29]	US	2020	unclear	492 days [IQR 76 – 996 days]	YES	857	222		74.1 ± 12.4	71.2 ± 12.8		530	109

**Table 2:** The number of deaths in each group and the number of major adverse events

Mortality within 30 days						LVOT obstruction			Stroke			Vascular complication		
All-cause mortality			Cardiovascular											
ViV	ViR	ViMAC	ViV	ViR	ViMAC	ViV	ViR	ViMAC	ViV	ViR	ViMAC	ViV	ViR	ViMAC
3	0	2	–	–	–	3	3	2	–	–	–	–	–	–
20	14	20	–	–	–	7	7	23	7	0	2	5	5	4
9/172	5/73	–	5/172	5/73	–	0/172	4/73	–	3/95	1/47	–	16/172	0/73	–
–	0	6	–	0	4	–	–	4	–	–	3	–	0	2
47/584	12/104	20	28/584	7/104	–	5	6	10	7	0	4	20	6	–
56	19	–	–	–	–	–	–	–	12	1	–	49	4	–

Acute kidney injury			Postprocedural Mitral Regurgitation											
			Trace/none			1 (+)			2 (+) or greater					
ViV	ViR	ViMAC	ViV	ViR	ViMAC	ViV	ViR	ViMAC	ViV	ViR	ViMAC			
–	–	–	–	–	–	–	–	–	–	–	–			
14	13	7	–	–	–	–	–	–	–	–	–			
7/172	4/73	–	107/145	40/67	–	30/145	19/67	–	8/145	8/67	–			
–	0	1	–	–	–	–	–	–	–	–	–			
–	–	–	557	63	55	93	44	36	8	3	2			
75	29	–	660	113	–	171	73	–	26	36	–

### Comparison of baseline characteristics

Before the quantitative meta-analysis, we conducted a comparative analysis of the baseline characteristics of the included studies. P(Z) marked in bold italic indicates that there was a difference, and italic indicates that there was no difference.

The main differences in the inclusion of the population in the ViV–ViR group were age (OR = 2.78, 95% CI: 1.23–3.72), proportion of females (OR = 1.73, 95% CI: 1.44–2.10), number of patients with atrial fibrillation (OR = 1.47, 95% CI: 1.18–1.84), creatinine value of patients (SMD = − 0.19, 95% CI: − 0.38–0.01), and number of patients with previous cerebrovascular accidents (OR = 1.59, 95% CI: 1.19–2.13). In addition, the creatinine value of the ViV group was lower than that of the ViR group (SMD = − 0.19, 95% CI: − 0.38 to − 0.01). For mode of progress failure, the patients in the ViV group showed more stenosis (OR = 2.69, 95% CI: 1.50–4.80) and combined (OR = 1.56, 95% CI: 1.22–1.99), while the patients in ViR group had more regurgitation (OR = 0.25, 95% CI: 0.16–0.40).

Similarly, we found some differences in the ViMAC-ViR group: the patients in the ViMAC group were 3.78 years older than those in the ViR group (95% CI: 0.91–6.66), and there were more women in the ViMAC group (OR = 2.21, 95% CI: 1.11–4.40) and patients with diabetes mellitus (OR = 1.60, 95% CI: 1.07–2.38). In addition, many patients in the ViMAC group had undergone TAVR surgery, which was 9.74 times that in the ViR group (95% CI: 2.54–37.36). However, the number of patients in the ViR group was greater than that in the ViMAC group, and the measured NT-proBNP value was also lower than that in the ViMAC group. These two values suggested that the heart condition of patients in the ViR group might be worse.

The results are shown in Tables 3 and 4 and Additional file 1: Figs. S1–S10.

**Table 3:** Baseline comparability analysis results (ViV vsViR)

Baseline	Studies	Effect measure	Model	Participants	Effect estimate	LCI	UCI	Q	P(Q)	Z	P(Z)
Age (years)	4	Mean difference	Fixed	1862	2.48	1.23	3.72	3.93	0.27	3.90	***< 0.01***
Female	5	Odds ratio	Fixed	2665	1.73	1.44	2.10	4.82	0.31	5.70	***< 0.01***
Diabetes mellitus	5	Odds Ratio	Fixed	2599	0.85	0.69	1.07	4.20	0.38	1.40	*0.16*
Hypertension	2	Odds ratio	Fixed	538	1.10	0.73	1.65	0.91	0.34	0.45	*0.65*
Atrial fibrillation	4	Odds ratio	Fixed	2136	1.47	1.18	1.84	3.83	0.28	3.45	***< 0.01***
Chronic kidney disease	3	Odds ratio	Random	2061	0.80	0.54	1.17	4.71	0.09	1.16	*0.25*
Severe/chronic lung disease	5	Odds ratio	Fixed	2599	1.00	0.81	1.24	2.70	0.61	0.01	*0.99*
Peripheral arterial disease	3	Odds ratio	Fixed	1353	0.84	0.60	1.18	0.41	0.82	1.01	*0.31*
Prior cerebrovascular accident	4	Odds ratio	Fixed	2420	1.59	1.19	2.13	0.57	0.90	3.12	***< 0.01***
Prior percutaneous coronary intervention	1	Odds ratio	Fixed	463	0.63	0.38	1.03	0.00	1.00	1.85	*0.06*
Prior pacemaker	2	Odds ratio	Random	1879	0.76	0.29	2.03	13.03	0.00	0.54	*0.59*
Prior aortic valve replacement	2										
TAVR	1	Odds ratio	Fixed	788	1.18	0.26	5.31	0.00	1.00	0.22	*0.83*
SAVR	2	Odds ratio	Fixed	960	1.15	0.74	1.78	0.00	0.99	0.62	*0.54*
STS risk score (%)	3	Mean difference	Random	630	4.88	− 1.65	11.40	22.13	0.00	1.47	*0.14*
Creatinine	2	Std. mean difference	Fixed	538	− 0.19	− 0.38	− 0.01	1.05	0.30	2.02	***0.04***
Hemoglobin (g/dl)	1	Mean difference	Fixed	75	0.60	− 0.84	2.04	0.00	1.00	0.82	*0.41*
NT-proBNP (ng/l)	1	Mean difference	Fixed	75	1006	− 1190	3202	0.00	1.00	0.90	*0.37*
Echocardiographic parameters	4										
LVEF, left ventricular ejection fraction (%)	4	Mean difference	Random	1862	11.01	9.49	12.53	4.68	0.20	14.17	***< 0.01***
Right ventricular systolic pressure (mm Hg)	1	Mean difference	Random	75	6.00	− 6.32	18.32	0.00	1.00	0.95	*0.34*
Mitral valve mean gradient (mmHg)	3	Mean difference	Random	1787	4.14	3.25	5.03	4.33	0.11	9.09	***< 0.01***
PASP (mmHg)	1	Mean difference	Random	1079	2.70	0.08	5.32	0.00	1.00	2.02	***0.04***
MVA, mitral valve area (cm^2^)	1	Mean difference	Random	1079	− 0.46	− 0.61	− 0.31	0.00	1.00	5.86	***< 0.01***
Mode of prosthesis failure	5										
Regurgitation	5	Odds ratio	Random	2630	0.25	0.16	0.40	10.17	0.04	5.89	***< 0.01***
Stenosis	5	Odds ratio	Random	2630	2.69	1.50	4.80	19.27	0.00	3.34	***< 0.01***
Combined	4	Odds ratio	Fixed	1827	1.56	1.22	1.99	1.82	0.61	3.52	***< 0.01***
NYHA	5										
I/II	2	Odds ratio	Random	1882	1.30	0.43	3.92	6.32	0.01	0.46	*0.64*
III/IV	5	Odds ratio	Random	2588	1.31	0.25	6.78	22.37	0.00	0.32	*0.75*

**Table 4:** Baseline comparability analysis results (ViMAC vs ViR)

Baseline	Studies	Effect measure	Model	Participants	Effect estimate	LCI	UCI	Q	P(Q)	Z	P(Z)
Age (years)	2	Mean difference	Fixed	226	3.78	0.91	6.66	1.17	0.28	2.58	***0.01***
Female	4	Odds ratio	Random	489	2.21	1.11	4.40	6.80	0.08	2.24	***0.02***
Diabetes mellitus	4	Odds ratio	Fixed	489	1.60	1.07	2.38	2.34	0.50	2.30	***0.02***
Hypertension	3	Odds ratio	Fixed	266	1.78	0.96	3.30	0.26	0.88	1.83	*0.07*
Atrial fibrillation	3	Odds ratio	Fixed	290	0.51	0.31	0.82	2.74	0.25	2.79	***0.01***
Chronic kidney disease	2	Odds ratio	Fixed	263	0.98	0.59	1.63	0.62	0.43	0.09	*0.93*
Severe/chronic lung disease	4	Odds ratio	Random	489	1.28	0.60	2.73	7.11	0.07	0.63	*0.53*
Peripheral Arterial disease	3	Odds ratio	Fixed	266	1.23	0.59	2.57	0.38	0.83	0.56	*0.57*
Prior cerebrovascular accident	4	Odds ratio	Fixed	489	1.24	0.74	2.06	5.46	0.14	0.81	*0.42*
Prior percutaneous coronary intervention	1	Odds ratio	Fixed	199	0.89	0.42	1.88	0.00	1.00	0.31	*0.76*
Prior pacemaker	1	Odds ratio	Fixed	223	0.98	0.53	1.81	0.00	0.00	0.07	*0.95*
Prior aortic valve Replacement	2										
TAVR	2	Odds ratio	Fixed	260	9.74	2.54	37.36	0.05	0.82	3.32	***< 0.01***
SAVR	2	Odds ratio	Random	262	1.11	0.19	6.45	5.40	0.02	0.11	*0.91*
STS risk score, %	2	Mean difference	Fixed	226	2.21	0.21	4.20	0.58	0.45	2.17	***0.03***
Creatinine	3	Std. mean difference	Fixed	266	0.06	− 0.20	0.33	0.45	0.80	0.48	*0.63*
Hemoglobin (g/dl)	2	Mean difference	Random	67	0.06	− 1.56	1.68	2.40	0.12	0.07	*0.94*
NT-proBNP (ng/l)	1	Mean difference	Fixed	27	− 2919	− 4825	− 1013	0.00	1.00	3.00	***< 0.01***
Echocardiographic parameters	3										
LVEF, left ventricular ejection fraction (%)	3	Mean difference	Fixed	266	13.01	9.77	16.25	0.34	0.84	7.88	***< 0.01***
Mitral valve mean gradient (mmHg)	1	Mean difference	Fixed	199	4.70	3.23	6.17	0.00	1.00	6.28	***< 0.01***
Right ventricular systolic pressure (mmHg)	1	Mean difference	Fixed	27	− 5.00	− 19.40	9.40	0.00	1.00	0.68	*0.50*
Mode of prosthesis failure	4										
Regurgitation	4	Odds ratio	Fixed	489	0.09	0.04	0.17	3.32	0.34	7.33	***< 0.01***
Stenosis	3	Odds ratio	Random	449	7.38	1.79	30.43	13.54	0.00	2.77	***0.01***
Combined	2	Odds ratio	Fixed	226	1.82	0.90	3.69	0.67	0.41	1.60	*0.10*
NYHA	4										
I/II	2	Odds ratio	Fixed	263	1.23	0.58	2.62	0.18	0.67	0.55	*0.58*
III/IV	4	Odds ratio	Fixed	489	1.02	0.56	1.84	0.48	0.79	0.06	*0.95*

### Meta-analysis of outcomes

#### ViV versus ViR

A total of five studies were included in this group. The ViV group included 2091 patients, and the ViR group included 574 patients. The following results were obtained by analysing the extracted data: the all-cause mortality within 30 days of the ViV group was lower than that of the ViR group, and the result was statistically significant [OR = 0.70, 95% CI (0.50–0.99), *P* = 0.04]. The left ventricular ejection fraction (LVEF) after the operation in the ViV group was significantly higher than that in the ViR group [MD = 8.74, 95% CI (7.03–10.46), *P* < 0.01], and the number of postprocedural trace/no mitral regurgitations was significantly higher than that in the ViR group [OR = 3.26, 95% CI (2.59 – 4.09), *P* < 0.01]. Life-threatening or fatal events [OR = 0.44, 95% CI (0.25–0.77), *P* < 0.01] and embolization [OR = 0.34, 95% CI (0.12–0.98), *P* = 0.05] were more likely to occur in the ViR group after surgery. Comparing the two groups, the probability of conversion to cardiac surgery in the ViR group [OR = 0.31, 95% CI (0.13–0.74), *P* = 0.01] and secondary valve implantation [OR = 0.21, 95% CI (0.13–0.33), *P* < 0.01] was also higher. At the same time, the VIR group was more prone to left ventricular outflow tract obstruction (LVOT) [OR = 0.22, 95% CI (0.11–0.44), *P* < 0.01]. The measured value of the mean Mitral valve gradient was slightly higher than that of the ViV group [MD = − 0.46, 95% CI (− 0.77–0.15), *P* < 0.01]. In addition, the incidence of acute kidney injury (AKI) in the VIR group increased [OR = 0.60, 95% CI (0.41–0.87), *P* < 0.01], postprocedural 1 (+) [OR = 0.45, 95% CI (0.35–0.00), *P* < 0.01] and 2 (+) or greater [OR = 0.22, 95% CI (0.14–0.35), *P* < 0.01]. Mitral regurgitation occurred more frequently. The other outcomes were not statistically significant, *P* > 0.05. The results are shown in Table 5 and Additional file 1: Figs. S10–S16.

**Table 5:** The results of meta-analysis of postoperative outcomes (ViV vs ViR)

Outcomes	Studies	Effect measure	Model	Participants	Effect estimate	LCI	UCI	Q	P(Q)	Z	P(Z)
All-cause Mortality within 30 days	5	Odds ratio	Fixed	2550	0.70	0.50	0.99	0.68	0.95	2.02	***0.04***
Cardiovascular	2	Odds ratio	Fixed	933	0.59	0.29	1.18	0.47	0.49	1.49	0.14
Noncardiovascular	1	Odds ratio	Fixed	688	0.67	0.24	1.82	0.00	1.00	0.79	0.43
Unknown-cause	4	Odds ratio	Fixed	1862	0.75	0.50	1.15	1.96	0.58	1.32	0.19
Bleeding	5	Odds ratio	Random	2665	0.68	0.13	3.61	73.19	0.00	0.45	0.65
Major or extensive bleeding	4	Odds ratio	Random	2420	1.66	0.98	2.80	3.11	0.38	1.89	0.06
Life-threatening or fatal	2	Odds ratio	Random	1266	0.44	0.25	0.77	0.48	0.49	2.85	***< 0.01***
Unknown	1	Odds ratio	Random	245	14.47	0.85	245.08	0.00	0.00	1.85	0.06
Embolization	4	Odds ratio	Fixed	1577	0.34	0.12	0.98	3.57	0.31	2.00	***0.045***
Conversion to Cardiac surgery	3	Odds ratio	Fixed	1341	0.31	0.13	0.74	2.27	0.32	2.64	***0.01***
Left ventricular outflow tract obstruction	4	Odds ratio	Fixed	1586	0.22	0.11	0.44	3.07	0.38	4.31	***< 0.01***
Stroke	4	Odds ratio	Fixed	2487	3.14	0.94	10.43	0.68	0.88	1.86	0.06
Post-procedural echocardiographic findings	4										
LVEF (%)	3	Mean difference	Fixed	1347	8.74	7.03	10.46	0.20	0.91	10.00	***< 0.01***
Mitral valve mean gradient (mmHg)	4	Mean difference	Fixed	1757	− 0.46	− 0.77	− 0.15	2.39	0.50	2.88	***< 0.01***
Right ventricular systolic pressure (mmHg)	1	Mean difference	Fixed	75	4.00	− 5.05	13.05	0.00	1.00	0.87	0.39
MVA, mitral valve area (cm^2^)	3	Mean difference	Fixed	1617	0.03	− 0.06	0.11	19.57	0.00	0.60	0.55
Vascular complication	4	Odds ratio	Random	2590	1.41	0.36	5.50	13.20	0.00	0.50	0.62
Acute kidney injury	3	Odds ratio	Fixed	1787	0.60	0.41	0.87	0.70	0.70	2.72	***0.01***
Need for second valve implantation	4	Odds ratio	Fixed	2420	0.21	0.14	0.33	0.70	0.87	7.13	***< 0.01***
Postprocedural mitral regurgitation	3										
Trace/none	3	Odds ratio	Fixed	2094	3.16	2.16	4.63	4.83	0.09	5.93	***< 0.01***
1 (+)	3	Odds ratio	Fixed	2094	0.44	0.28	0.70	6.17	0.05	3.47	***< 0.01***
2 (+) or greater	3	Odds ratio	Fixed	2094	0.27	0.13	0.59	4.23	0.12	3.28	***< 0.01***

### ViMAC versus ViR

A total of four studies were included in this group, of which ViMAC included 198 patients and ViR included 291 patients. After analysing the data by RevMan 5.4, the results of all-cause mortality within 30 days [OR = 2.95, 95% CI (1.76–4.93), *P* < 0.01], stroke [OR = 5.16, 95% CI (1.14–23.38), *P* = 0.03] and LVEF [MD = 13.64, 95% CI (10.02–17.26), *P* < 0.01] of the ViMAC group were all significantly higher than those of the ViR group, *P* < 0.05. The other outcomes were not statistically significant, *P* > 0.05. The results are shown in Table 6 and Additional file 1: Figs. S17–S21.

**Table 6:** The results of meta-analysis of postoperative outcomes (ViMAC vs ViR)

Outcomes	Studies	Effect measure	Model	Participants	Effect estimate	LCI	UCI	Q	P(Q)	Z	P(Z)
All-cause Mortality within 30 days	4	Odds ratio	Fixed	470	3.20	1.89	5.43	3.27	0.35	4.32	***< 0.01***
Cardiovascular	2	Odds ratio	Fixed	244	1.96	0.78	4.96	0.38	0.54	1.43	0.15
Noncardiovascular	2	Odds ratio	Fixed	244	2.01	0.70	5.80	0.01	0.91	1.29	0.20
Unknown-cause	2	odds ratio	Fixed	226	4.94	2.33	10.46	0.07	0.79	4.17	***< 0.01***
Bleeding	4	Odds ratio	Random	489	0.62	0.17	2.17	9.72	0.02	0.75	0.45
Minor	1	Odds ratio	Fixed	40	2.36	0.11	52.88	0.00	1.00	0.54	0.59
Major or extensive bleeding	4	Odds ratio	Fixed	489	1.20	0.43	3.35	1.22	0.75	0.35	0.73
Life-threatening or fatal	3	Odds ratio	Fixed	462	0.76	0.36	1.60	0.44	0.80	0.73	0.46
Embolization	4	Odds ratio	Fixed	489	1.92	0.71	5.19	2.46	0.48	1.29	0.20
Conversion to Cardiac surgery	3	Odds ratio	Fixed	449	1.38	0.54	3.53	3.02	0.22	0.67	0.51
Left ventricular outflow tract obstruction	4	Odds ratio	Random	489	3.02	0.82	11.05	10.14	0.02	1.67	0.10
Stroke	3	Odds ratio	Fixed	462	5.16	1.14	23.38	1.88	0.39	2.13	***0.03***
Post-procedural echocardiographic findings											
LVEF (%)	2	Mean difference	Fixed	226	13.64	10.02	17.26	0.00	0.95	7.39	***< 0.01***
Mitral valve mean gradient (mmHg)	2	Mean difference	Fixed	226	− 1.54	− 2.38	− 0.71	1.14	0.28	3.62	***< 0.01***
Right ventricular systolic pressure (mmHg)	1	Mean difference	Fixed	27	− 1.00	− 13.57	11.57	0.00	1.00	0.16	0.88
MVA, mitral valve area (cm^2^)	2	Mean difference	Fixed	226	0.65	0.36	0.93	1.01	0.31	4.45	***< 0.01***
Vascular complication	3	Odds ratio	Fixed	462	1.31	0.54	3.16	1.05	0.59	0.60	0.55
Acute kidney injury	2	Odds ratio	Fixed	239	1.35	0.53	3.44	0.00	1.00	0.63	0.53
Need for second valve implantation	3	Odds ratio	Random	449	1.06	0.34	3.31	4.41	0.11	0.09	0.92
Postprocedural mitral regurgitation											
Trace/none	1	Odds ratio	Fixed	223	1.16	0.69	1.98	0.00	1.00	0.56	0.57
1 (+)	1	Odds ratio	Fixed	223	1.01	0.58	1.75	0.00	1.00	0.04	0.97
2 (+) or greater	1	Odds ratio	Fixed	223	0.82	0.13	4.98	0.00	1.00	0.22	0.83

### Indirect comparison

**Fig. 3:**
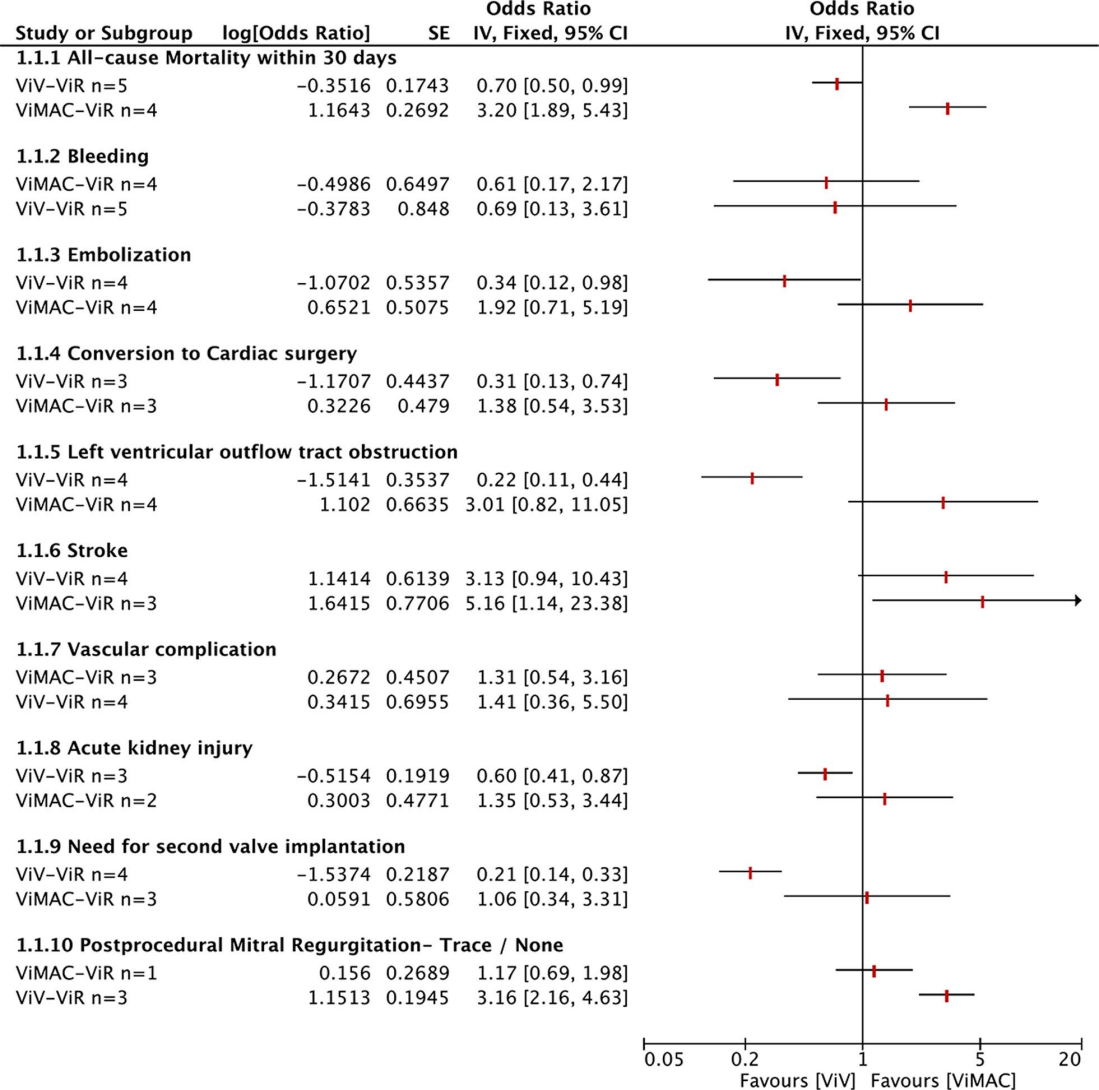
The group of ViV was indirectly compared with the ViMAC based on the ViR group

According to the different analysis results of the ViV–ViR and ViMAC–ViR groups, we further conducted an indirect comparative analysis between the TMViV and TMViMAC groups. Compared with the ViR group, the all-cause mortality within 30 days of the ViV group decreased by 0.3. The probability of embolization, LVOT obstruction, conversion to cardiac surgery and need for second valve implantations decreased by 0.66, 0.78, 0.69 and 0.79, respectively. Mortality in the ViMAC group increased by 2.20, and the other probabilities increased by 0.92, 2.01, 0.38 and 0.06 (*P* < 0.05). Regarding postoperative trace/no mitral regurgitation, there were more in the ViV and ViMAC groups than the ViR group. According to the statistical analysis results, they were 2.16 and 0.17 higher than the ViR, respectively. No significant statistical results were found in outcome indicators such as blending, stroke, vascular composition or acute kidney injury. The results are shown in Fig. 3.

### Publication bias

Since only six articles (< 10) were included in the final collection, we did not evaluate publication bias [30].

## Discussion

Transcatheter mitral valve replacement (TMVR) seems to have become a viable option for patients with severe mitral valve disease, such as severe mitral valve bioprosthesis degradation, failure of valvuloplasty or severe mitral valve natural annulus calcification. Moreover, a certain clinical effect can be obtained, and the probability of complications has also been declining with the progress of surgical methods and experience of doctors [14]. We conducted this comprehensive and systematic review and meta-analysis to promote and disseminate these technologies in clinical diagnosis and treatment and enhance their value.

In this meta-analysis, we found that some of the baseline information in the ViV–ViR group was incomparable, such as age, atrial fibrillation and previous cerebrovascular accidents. However, when analysing the impact of this baseline information on these two groups, the differences indicated that the situation of the ViV group was worse. The average age of patients in the ViV group was 2.48 years older than that in the ViR group, and the number of patients in the ViV group with atrial fibrillation was 1.47 times that of the ViR group. Nevertheless, TMViV still showed good applicability. The all-cause mortality within 30 days of the ViV group was lower than that of the ViR group, which was also proven in many large sample studies [20, 25, 28]. In addition, the analysis results indicated that the incidence of acute kidney injury in the ViR group was significantly greater than that in the ViV group. However, in terms of all-cause mortality, Yoon [20] and Matheus Simonato's [29] prediction analysis of death factors showed that the two most important factors were "TMViR" and "Chronic Kidney Disease". In addition, the ViV group also showed better results in other clinical outcomes, such as a smaller probability of nonfatal haemorrhage and lower incidence of left ventricular outflow tract obstruction and embolism. Moreover, the probability of the ViV group being converted to cardiac surgery due to accidents during the treatment process was also less than that of the ViR group, which was more in line with our original intention of using a transcatheter for revalvular surgery. At the same time, the possibility of high-grade mitral valve postoperative residual regurgitation in the ViV group was far less than that in the ViR group. In contrast, it was more likely to be trace/none. This also proved the feasibility of transcatheter surgery in patients with mitral valve bioprostheses (TMViVs).

Compared with TMViR, TMViMAC had higher short-term mortality when applied to patients with severe MAC. The same conclusion was reached in some multicentre joint studies. Among the patients with high surgical risk (advanced age or higher risk score of the Society of Thoracic Surgeons, etc.), the 30-day all-cause mortality of TMViMAC was very high. Patients at special risk, such as patients with chronic lung disease, chronic renal insufficiency and high ejection fraction, might require multiple valve operations [31–34]. From the comparison of baseline information, age might be a potential explanation for the higher mortality rate in the ViMAC group. However, the higher number of atrial fibrillations (ViMAC vs. ViR: OR = 0.51) in the ViR group and the higher probability of heart failure (NT-proBNP, ViMAC vs. ViR: MD < 0) indicated that the situation of the ViR group was worse. Although the results of LVOT obstruction in the ViR-ViMAC group obtained by this meta-analysis were not statistically significant (*P* = 0.10), the incidence of patients in the ViMAC group was significantly higher than that in the ViR group. Coupled with the lower incidence of stroke in the ViR group after surgery, we had more reason to believe that TMViR was better than TMViMAC.

Based on the ViR group, through indirect analysis, the ViV group had the lowest all-cause mortality within 30 days, which might benefit from fewer complications and a lower incidence of residual mitral regurgitation. The lower the incidence of LVOT obstruction was, the smaller the possibility of switching to traditional heart surgery and replacing the valve again. All this evidence allowed us to see its excellent effects. It was suggested that TMViV might become the first-line treatment method for the treatment of mitral valve bioprostheses. Regarding the high incidence of embolization in the three groups, long-term postoperative anticoagulation therapy might be a better approach [35]. For some relatively poor outcomes of ViMAC, existing studies believe that this might be related to more basic information and comorbidities, such as age, sex, diabetes and renal impairment [36–38]. However, a more decisive factor might be the baseline CT-MAC calcium score [36]. The higher the patient's MAC baseline was, the higher the disease activity and the faster the progression. It also reflected the vicious calcium cycle that was established in the patient's body, which further calcified the mitral valve. Even so, TMVR is still a viable alternative treatment option for some inoperable severe MAC patients [27, 31, 32]. Perhaps surgical mitral valve replacement utilizing a transcatheter aortic valve in the mitral position (MVR–TAVR) used by Joseph would be another viable option for patients with severe MAC [39].

At present, the better applicability of TMViV, TMViR and TMViMAC has been proven by many studies, including some multicentre clinical research results [25, 28, 29, 32]. It was undeniable that these operations still had some common serious complications, such as LVOT obstruction, bleeding and acute kidney injury. Although there were various difficulties, scholars in various countries were actively looking for solutions, for instance, improving postoperative outcomes through surgical approaches. In a study by Eleid [40], transfemoral percutaneous venous mitral valve implantation in patients with a high risk of bioprosthesis degradation was safe and effective. It was also conducive to rapid improvement of haemodynamics and functional status. For patients with annuloplasty ring failure and severe MAC, further studies are needed in view of the high short-term morbidity and mortality feasibility. Transseptal TMVR, which eliminates the need for extracorporeal circulation and naturally reduces the risk, is considered a safer route to perform repeat valve surgery [19, 41]. It has the advantages of less invasiveness, does not require opening the chest and avoids trauma to the left ventricle. Because of this, it is more popular with patients and clinicians [42]. In addition, in terms of the Achilles heel of TMVR, there have been increasing studies on iatrogenic LVOT obstruction, which was defined as an LVOT peak gradient increase of ≥ 10 mmHg post-TMVR. [43–46]. The incidence of LVOT obstruction in TMVR occurs in up to 10–40% of ViMAC, 5% of ViR, and 0.7–2% of ViV cases [20, 28]. Once it happens, the result will be very poor, and the hospital mortality rate may be as high as 62% [19]. Therefore, it was particularly important to study how to solve or avoid this problem. It is worth emphasizing that preventing the risk of LVOT obstruction was the key to improving the results. Among the published methods, the intentional transcatheter laceration of the anterior mitral valve leaflet (LAMPOON) technique and alcohol septal ablation (ASA) are considered effective [45, 47]. At the same time, it has also been well proven in the research of Khan [48] that LAMPOON could effectively prevent LVOT obstruction from TMVR. However, neither of these two technologies has been approved by the Food and Drug Administration (FDA). Perhaps in the future, large sample data could provide different results.

Although a more detailed comparison and analysis were performed, there were still certain limitations. First, there were few studies available for analysis, especially the study on TMViMAC, and the accuracy of the results would be affected. Second, due to the follow-up time and content of different studies, we only obtained an early result. However, the overall quality of the included literature was relatively high, and the results were relatively reliable. Third, some baselines were incomparable, but the baseline information all had a positive impact, which was opposite to the direction of the outcome indicators. Such results suggest that better patient baseline information will increase the credibility of the outcome. In addition, there was a lack of evidence for a direct comparison between TMViV and TMViMAC in the included studies, but we conducted an indirect comparison this time to answer the pros and cons of these two procedures. Considering these limitations, the results of this meta-analysis need to be interpreted carefully, and we look forward to better randomizing clinical trial comparison models in the future to further prove the feasibility of TMViV, TMViR and TMViMAC.

## Conclusion

In summary, the existing evidence shows that TMViV and TMViMAC have lower mortality and complication rates, which are favoured by many patients. TMVR showed promise for patients with severe MAC, and further studies are needed to prove its feasibility.

## Supplementary Information

**Additional file 1: Fig. S1.** Comparison of baseline information between the group of ViV and ViR. The type of data analyzed was Dichotomous using the Random effects. **Fig. S2.** Comparison of baseline information between the group of ViV and ViR. The type of data analyzed was Dichotomous using the Fixed effects. **Fig. S3**. Comparison of baseline information between the group of ViV and ViR. The type of data analyzed was Continuous (MD) using the Random effects. **Fig. S4.** Comparison of baseline information between the group of ViV and ViR. The type of data analyzed was Continuous (MD) using the Fixed effects. **Fig. S5**. Comparison of baseline information between the group of ViV and ViR. The type of data analyzed was Continuous (SMD) using the Fixed effects. **Fig. S6**. Comparison of baseline information between the group of ViMAC and ViR. The type of data analyzed was Dichotomous using the Random effects. **Fig. S7**. Comparison of baseline information between the group of ViMAC and ViR. The type of data analyzed was Dichotomous using the Fixed effects. **Fig. S8**. Comparison of baseline information between the group of ViMAC and ViR. The type of data analyzed was Continuous (MD) using the Random effects. **Fig. S9**. Comparison of baseline information between the group of ViMAC and ViR. The type of data analyzed was Continuous (MD) using the Fixed effects. **Fig. S10**. Comparison of baseline information between the group of ViMAC and ViR. The type of data analyzed was Continuous (SMD) using the Fixed effects. **Fig. S11**. Comparison of Outcomes between the group of ViV and ViR. The type of data analyzed was Dichotomous using the Random effects. **Fig. S12**. Subgroup of Bleeding between the group of ViV and ViR. The type of data analyzed was Dichotomous using the Random effects. **Fig. S13**. Comparison of Outcomes between the group of ViV and ViR. The type of data analyzed was Dichotomous using the Fixed effects. **Fig. S14**. Subgroup of all-cause mortality within 30 days between the group of ViV and ViR. The type of data analyzed was Dichotomous using the Fixed effects. **Fig. S15**. Comparison of Outcomes between the group of ViV and ViR. The type of data analyzed was Continuous (MD) using the Fixed effects. **Fig. S16**. Comparison of Outcomes between the group of ViV and ViR. The type of data analyzed was Continuous (SMD) using the Fixed effects. **Fig. S17**. Comparison of Outcomes between the group of ViMAC and ViR. The type of data analyzed was Dichotomous using the Random effects. **Fig. S18**. Subgroup of Bleeding between the group of ViMAC and ViR. The type of data analyzed was Dichotomous using the Fixed effects. **Fig. S19**. Comparison of Outcomes between the group of ViMAC and ViR. The type of data analyzed was Dichotomous using the Fixed effects. **Fig. S20**. Subgroup of all-cause mortality within 30 days between the group of ViMAC and ViR. The type of data analyzed was Dichotomous using the Fixed effects. **Fig. S21**. Comparison of Outcomes between the group of ViMAC and ViR. The type of data analyzed was Continuous (MD) using the Fixed effects.

## Data Availability

All data generated or analysed during this study are included in this published article and its supplementary information files.

## Abbreviations

ViV: Valve-in-valve
ViR: Valve-in-ring
ViMAC: Valve-in-MAC
MAC: Mitral annulus calcification
AHA: American Heart Association
TMVR: Transcatheter mitral valve replacement
TMViV: Transcatheter mitral valve-in-valve
TMViR: Transcatheter mitral valve-in-ring
TMViMAC: Transcatheter mitral valve-in-MAC
NOS: Newcastle–Ottawa Scale
OR: Odds ratio
MD: Mean difference
SMD: Std. mean difference
LVEF: Left ventricular ejection fraction
LVOT: Left ventricular outflow tract
TAVR: Transcatheter aortic valve replacement
SAVR: Surgical aortic valve replacement
MVR-TAVR: Mitral valve replacement utilizing a transcatheter aortic valve in the mitral position
FDA: Food and Drug Administration
