# Supplementary material for: Transcatheter mitral valve replacement for degenerated mitral valve bioprostheses, failure of mitral valvuloplasty and native valve with severe mitral annulus calcification: a systematic review and meta-analysis
Source: J Cardiothorac Surg. 2021 Oct 10;16:293. doi: 10.1186/s13019-021-01677-7 (PMC8504093; doi:10.1186/s13019-021-01677-7)

***Transcatheter Mitral Valve Replacement for Degenerated Mitral Valve Bioprostheses, Failure of Mitral Valvuloplasty and Native Valve with Severe Mitral Annulus Calcification: A Systematic Review and Meta-Analysis***

***Description of Supplementary Figures：***

Figure S1 Comparison of baseline information between the group of ViV and ViR. The type of data analyzed was Dichotomous using the Random effects.

Figure S2 Comparison of baseline information between the group of ViV and ViR. The type of data analyzed was Dichotomous using the Fixed effects.

Figure S3 Comparison of baseline information between the group of ViV and ViR. The type of data analyzed was Continuous (MD) using the Random effects.

Figure S4 Comparison of baseline information between the group of ViV and ViR. The type of data analyzed was Continuous (MD) using the Fixed effects.

Figure S5 Comparison of baseline information between the group of ViV and ViR. The type of data analyzed was Continuous (SMD) using the Fixed effects.

Figure S6 Comparison of baseline information between the group of ViMAC and ViR. The type of data analyzed was Dichotomous using the Random effects.

Figure S7 Comparison of baseline information between the group of ViMAC and ViR. The type of data analyzed was Dichotomous using the Fixed effects.

Figure S8 Comparison of baseline information between the group of ViMAC and ViR. The type of data analyzed was Continuous (MD) using the Random effects.

Figure S9 Comparison of baseline information between the group of ViMAC and ViR. The type of data analyzed was Continuous (MD) using the Fixed effects.

Figure S10 Comparison of baseline information between the group of ViMAC and ViR. The type of data analyzed was Continuous (SMD) using the Fixed effects.

Figure S11 Comparison of Outcomes between the group of ViV and ViR. The type of data analyzed was Dichotomous using the Random effects.

Figure S12 Subgroup of Bleeding between the group of ViV and ViR. The type of data analyzed was Dichotomous using the Random effects.

Figure S13 Comparison of Outcomes between the group of ViV and ViR. The type of data analyzed was Dichotomous using the Fixed effects.

Figure S14 Subgroup of all-cause mortality within 30 days between the group of ViV and ViR. The type of data analyzed was Dichotomous using the Fixed effects.

Figure S15 Comparison of Outcomes between the group of ViV and ViR. The type of data analyzed was Continuous (MD) using the Fixed effects.

Figure S16 Comparison of Outcomes between the group of ViV and ViR. The type of data analyzed was Continuous (SMD) using the Fixed effects.

Figure S17 Comparison of Outcomes between the group of ViMAC and ViR. The type of data analyzed was Dichotomous using the Random effects.

Figure S18 Subgroup of Bleeding between the group of ViMAC and ViR. The type of data analyzed was Dichotomous using the Fixed effects.

Figure S19 Comparison of Outcomes between the group of ViMAC and ViR. The type of data analyzed was Dichotomous using the Fixed effects.

Figure S20 Subgroup of all-cause mortality within 30 days between the group of ViMAC and ViR. The type of data analyzed was Dichotomous using the Fixed effects.

Figure S21 Comparison of Outcomes between the group of ViMAC and ViR. The type of data analyzed was Continuous (MD) using the Fixed effects.

Figure S1 Comparison of baseline information between the group of ViV and ViR. The type of data analyzed was Dichotomous using the Random effects.


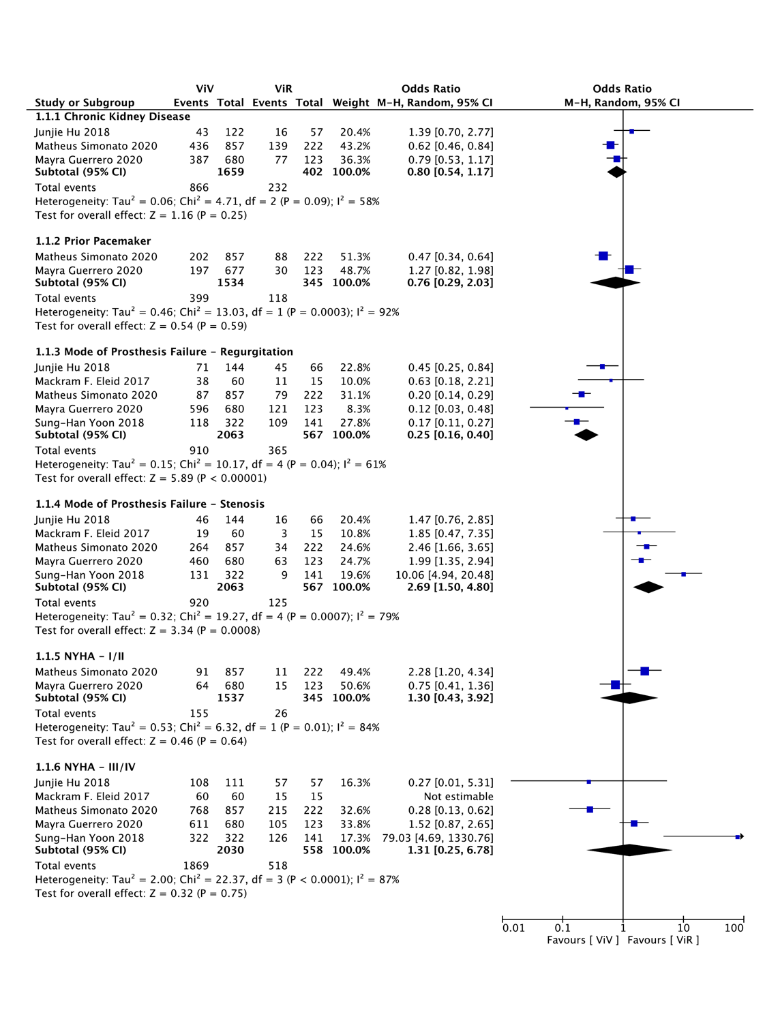


Figure S2 Comparison of baseline information between the group of ViV and ViR. The type of data analyzed was Dichotomous using the Fixed effects.


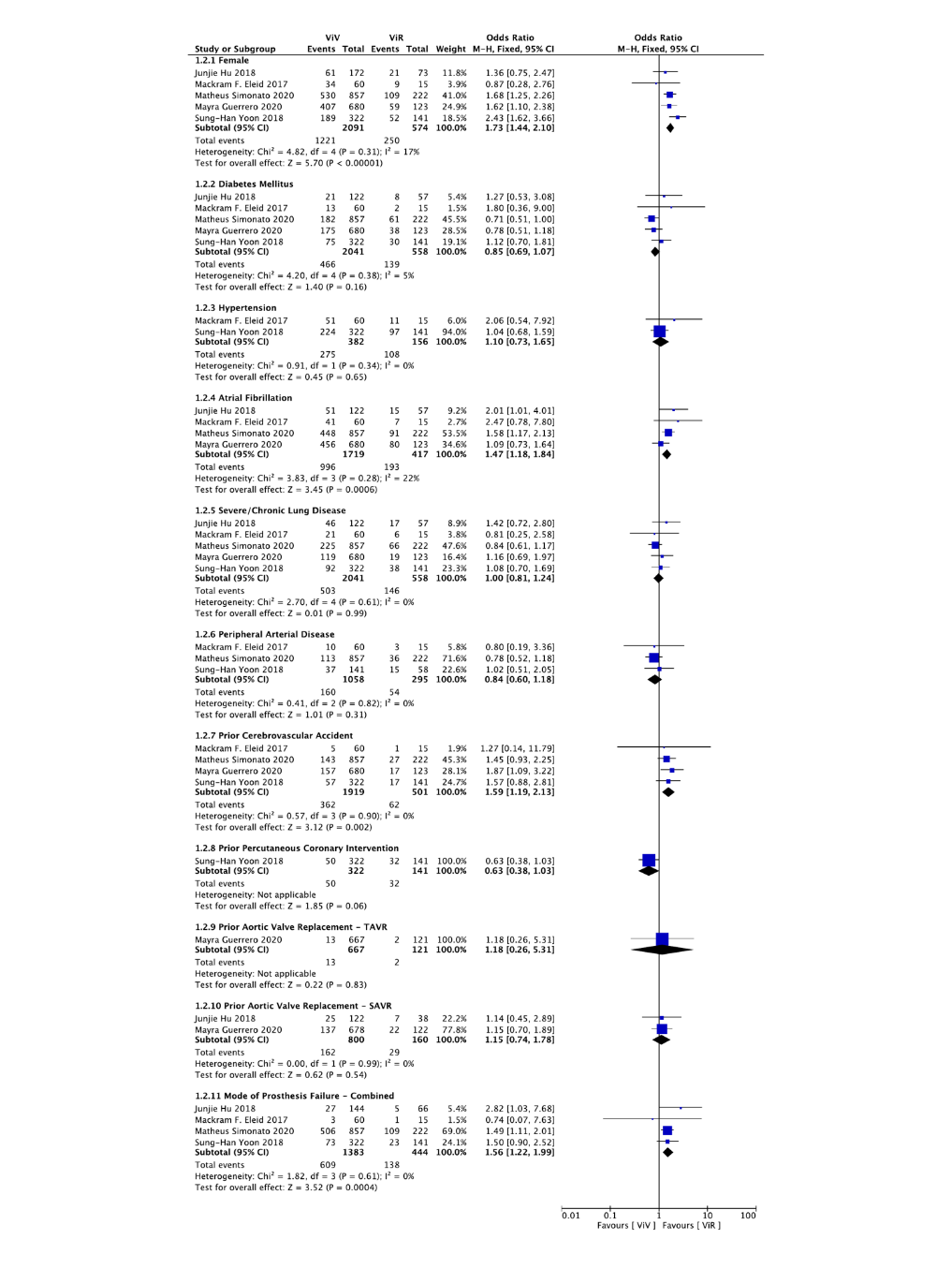


Figure S3 Comparison of baseline information between the group of ViV and ViR. The type of data analyzed was Continuous (MD) using the Random effects.


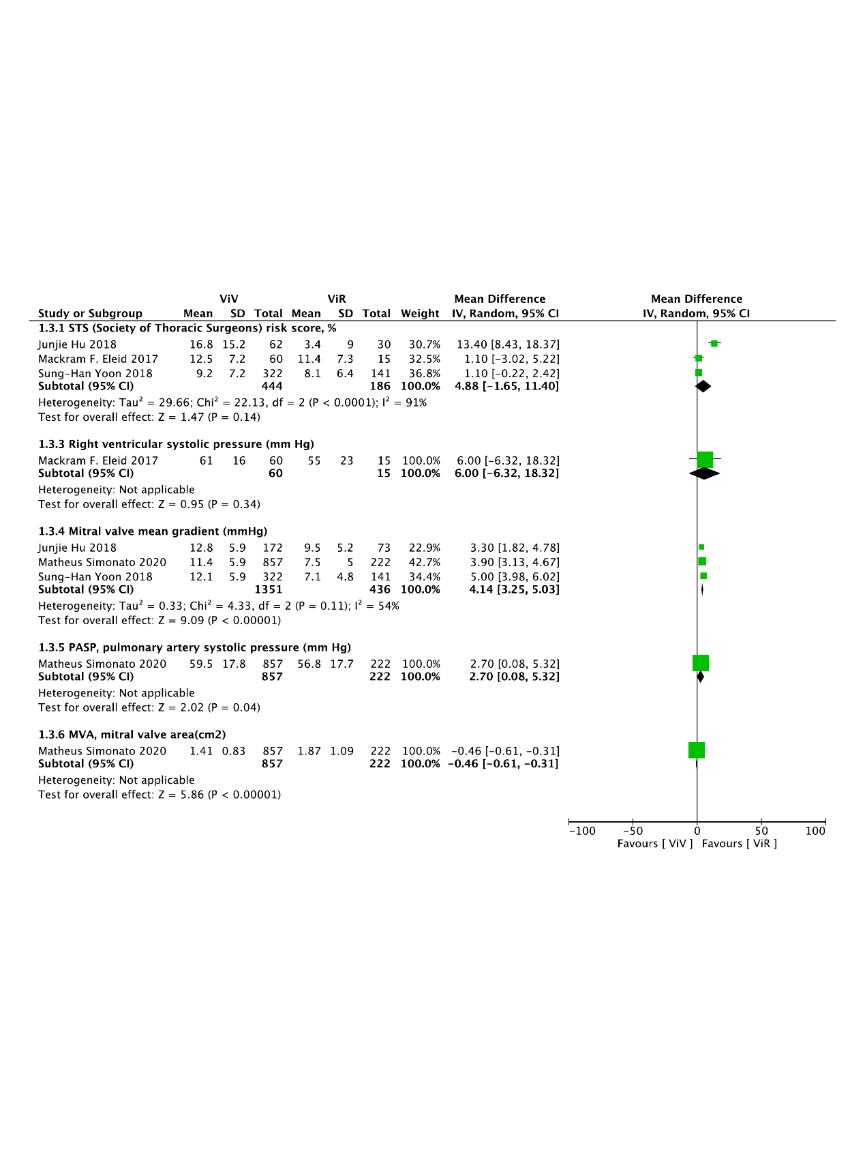


Figure S4 Comparison of baseline information between the group of ViV and ViR. The type of data analyzed was Continuous (MD) using the Fixed effects.


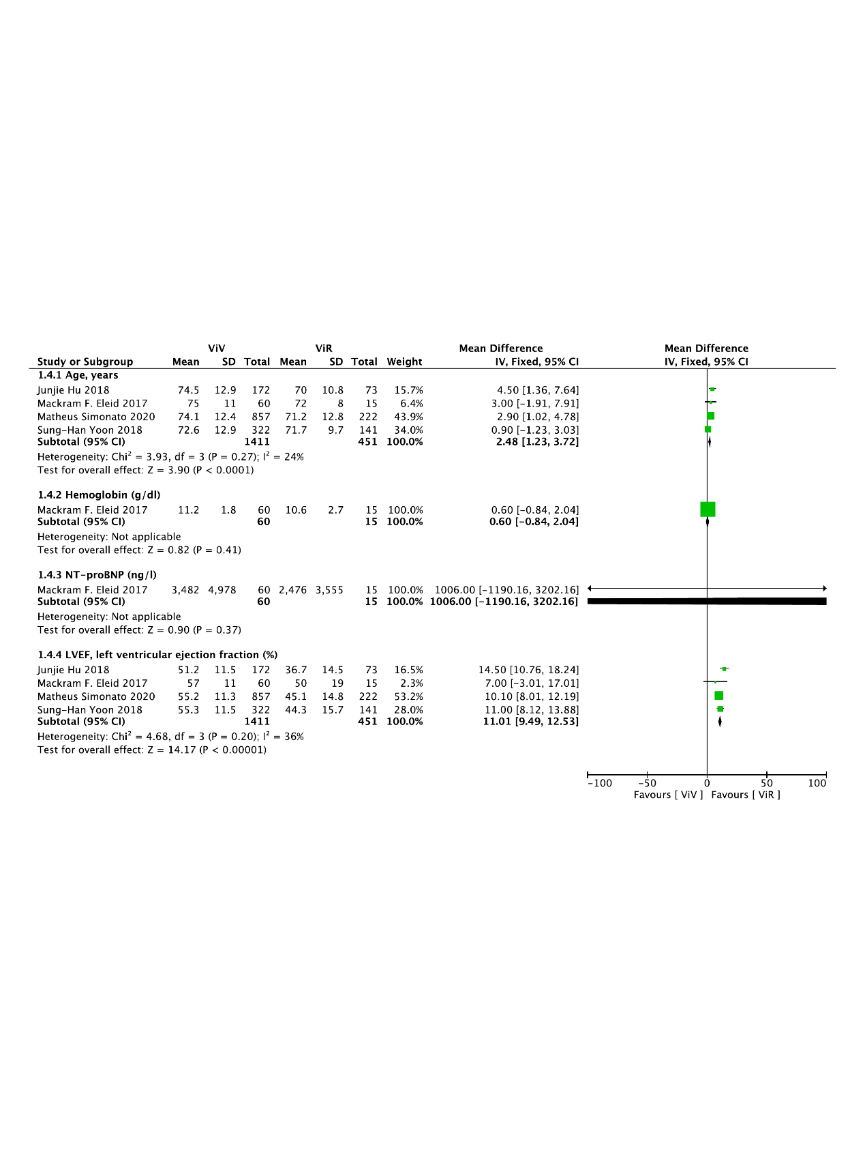


Figure S5 Comparison of baseline information between the group of ViV and ViR. The type of data analyzed was Continuous (SMD) using the Fixed effects.


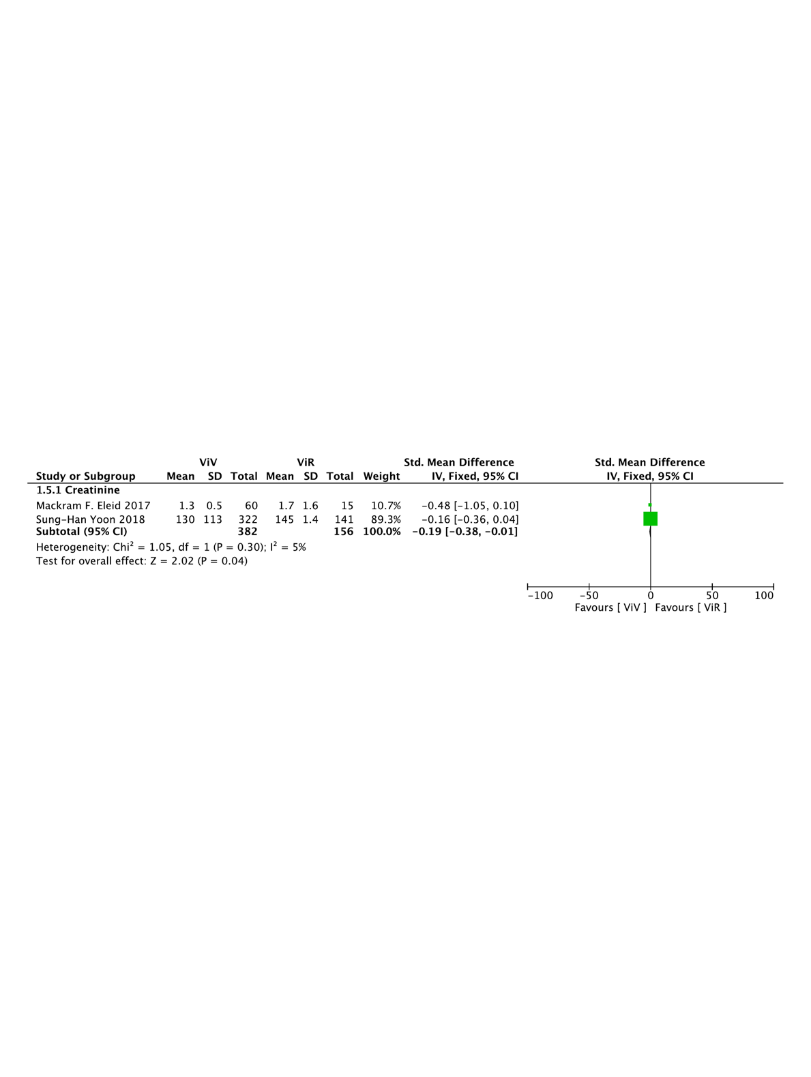


Figure S6 Comparison of baseline information between the group of ViMAC and ViR. The type of data analyzed was Dichotomous using the Random effects.


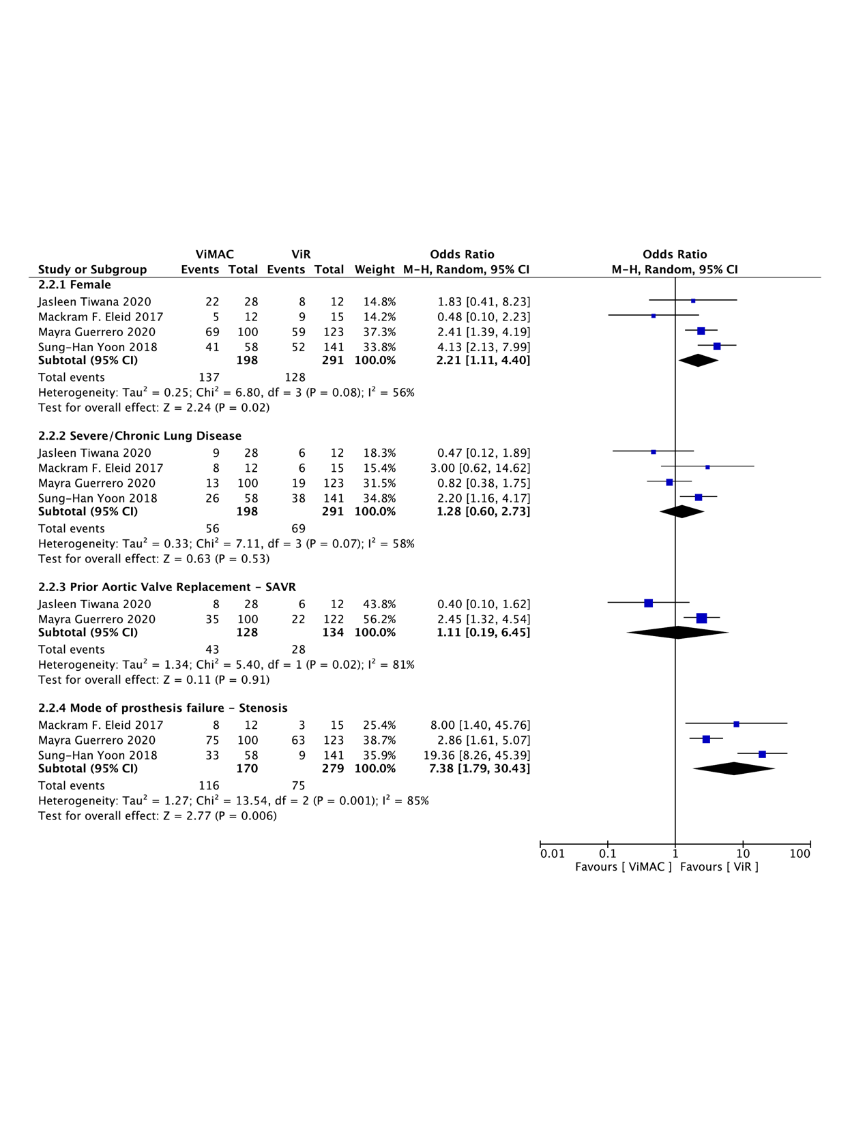


Figure S7 Comparison of baseline information between the group of ViMAC and ViR. The type of data analyzed was Dichotomous using the Fixed effects.


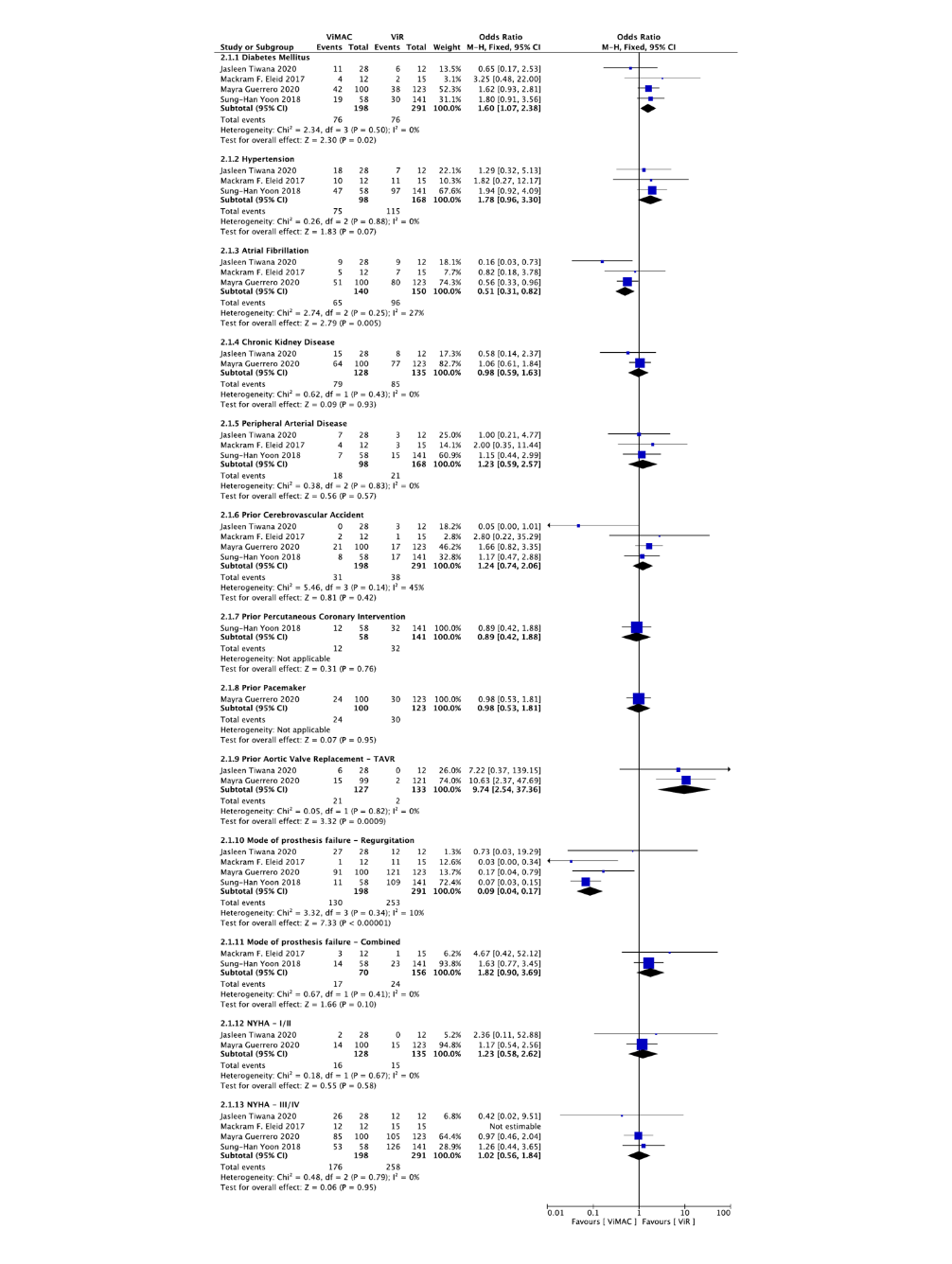


Figure S8 Comparison of baseline information between the group of ViMAC and ViR. The type of data analyzed was Continuous (MD) using the Random effects.


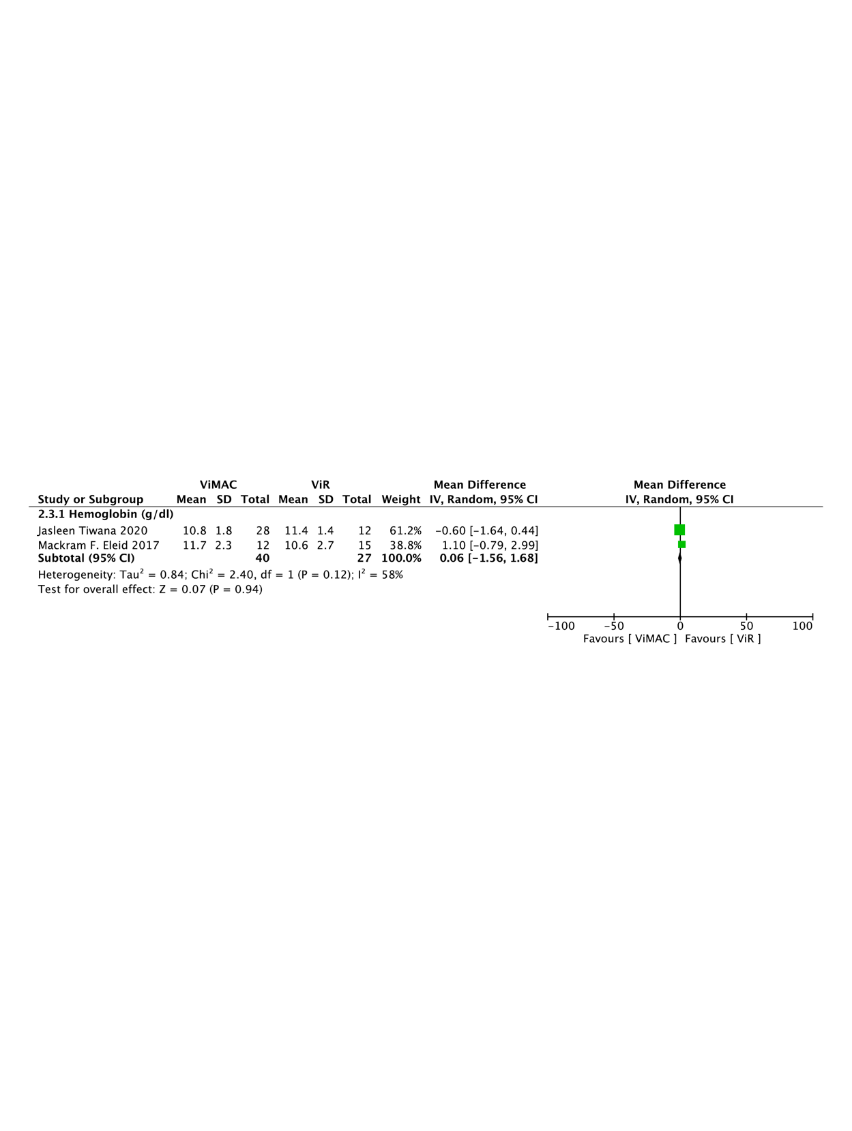


Figure S9 Comparison of baseline information between the group of ViMAC and ViR. The type of data analyzed was Continuous (MD) using the Fixed effects.


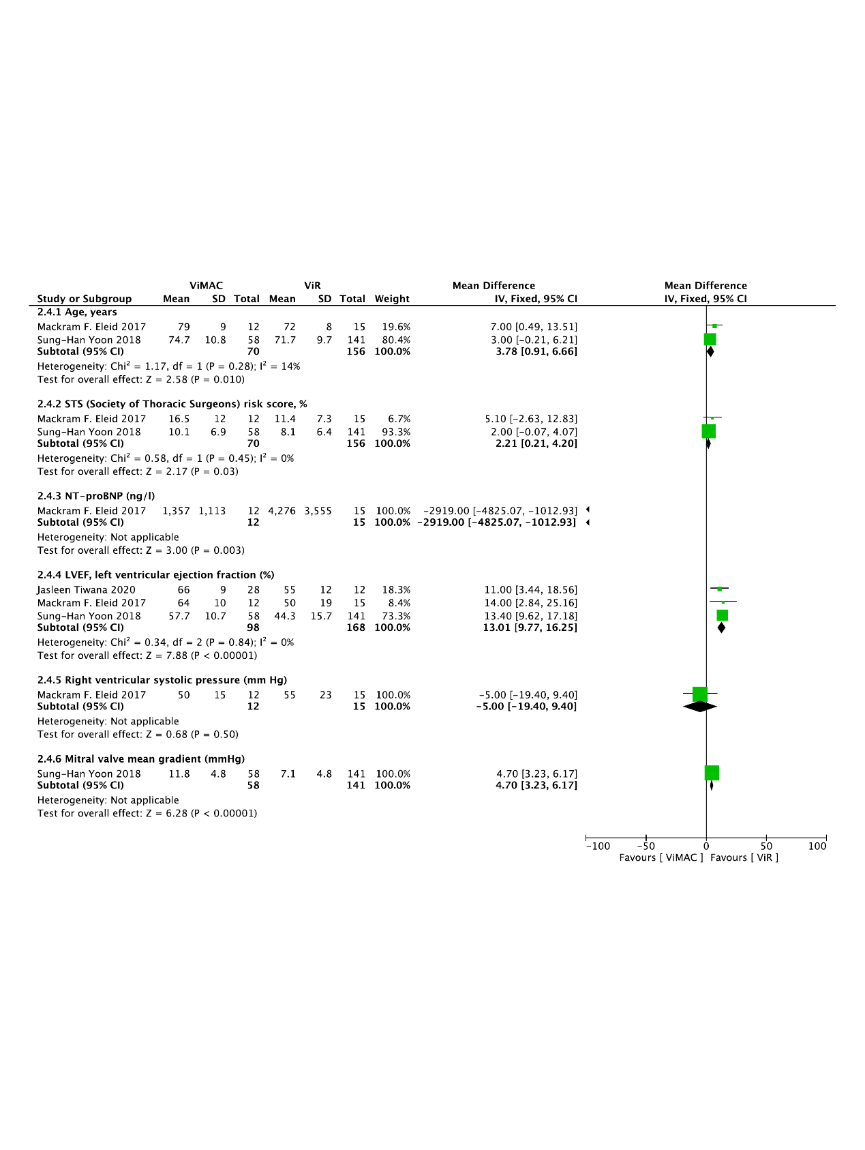


Figure S10 Comparison of baseline information between the group of ViMAC and ViR. The type of data analyzed was Continuous (SMD) using the Fixed effects.


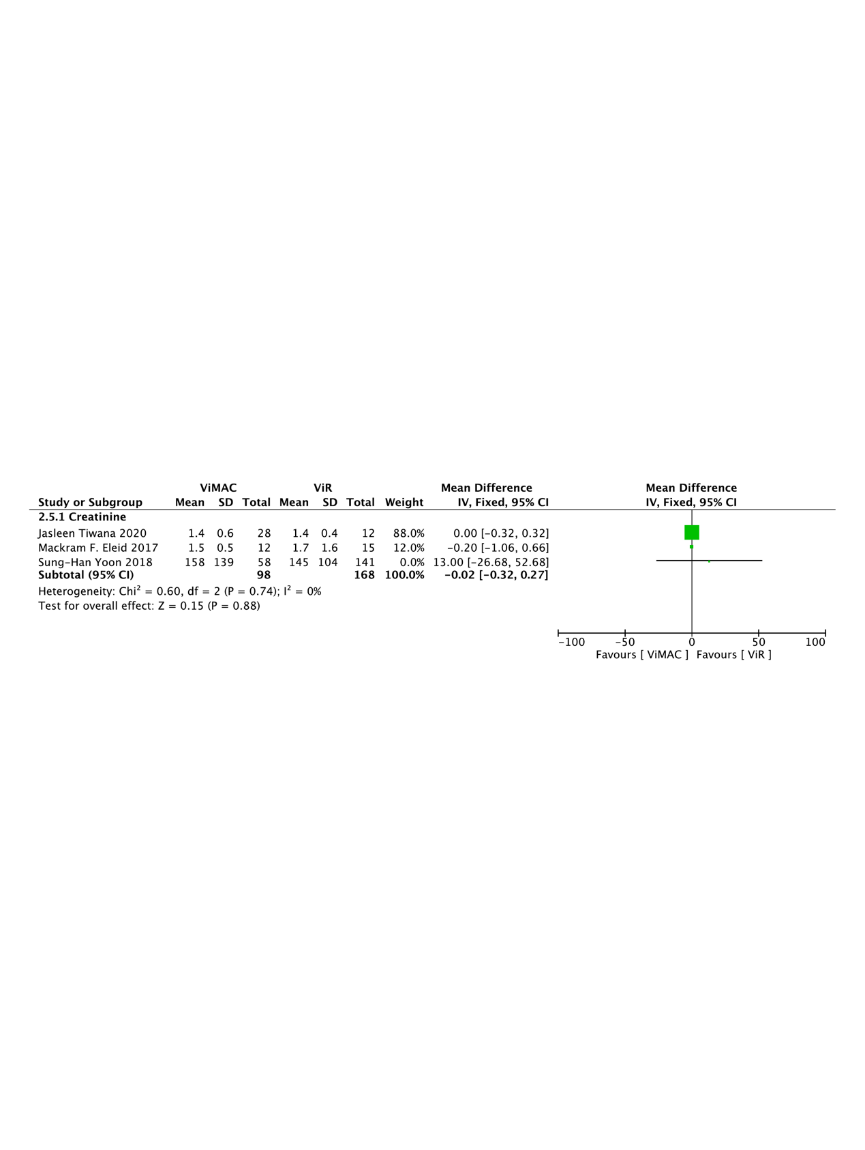


Figure S11 Comparison of Outcomes between the group of ViV and ViR. The type of data analyzed was Dichotomous using the Random effects.


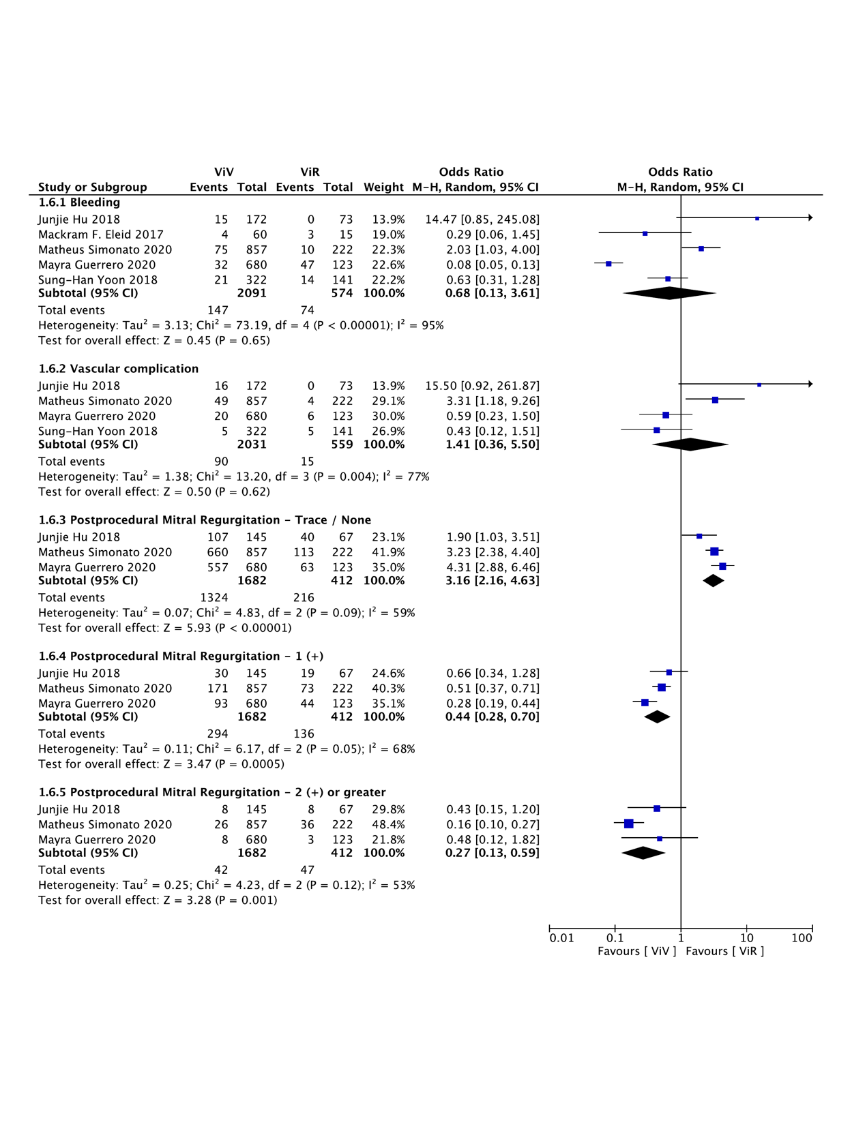


Figure S12 Subgroup of Bleeding between the group of ViV and ViR. The type of data analyzed was Dichotomous using the Random effects.


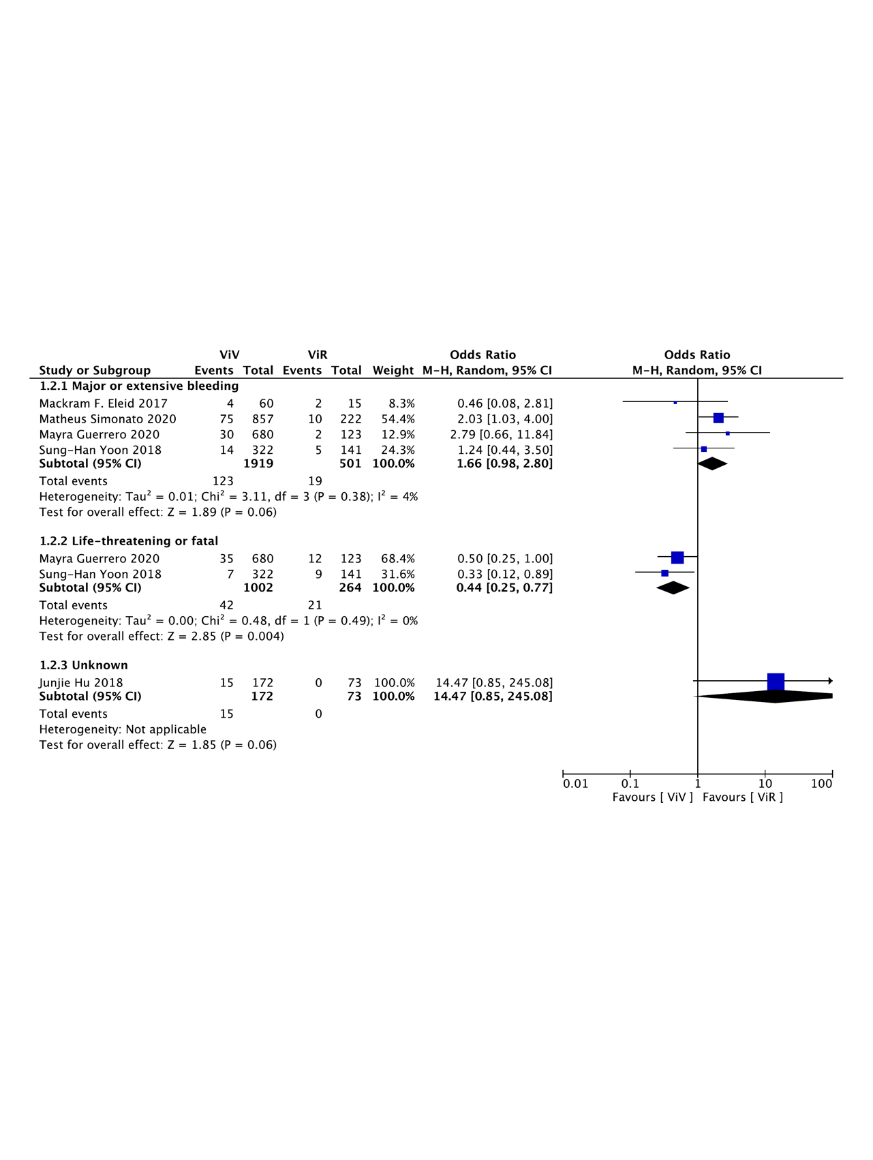


Figure S13 Comparison of Outcomes between the group of ViV and ViR. The type of data analyzed was Dichotomous using the Fixed effects.


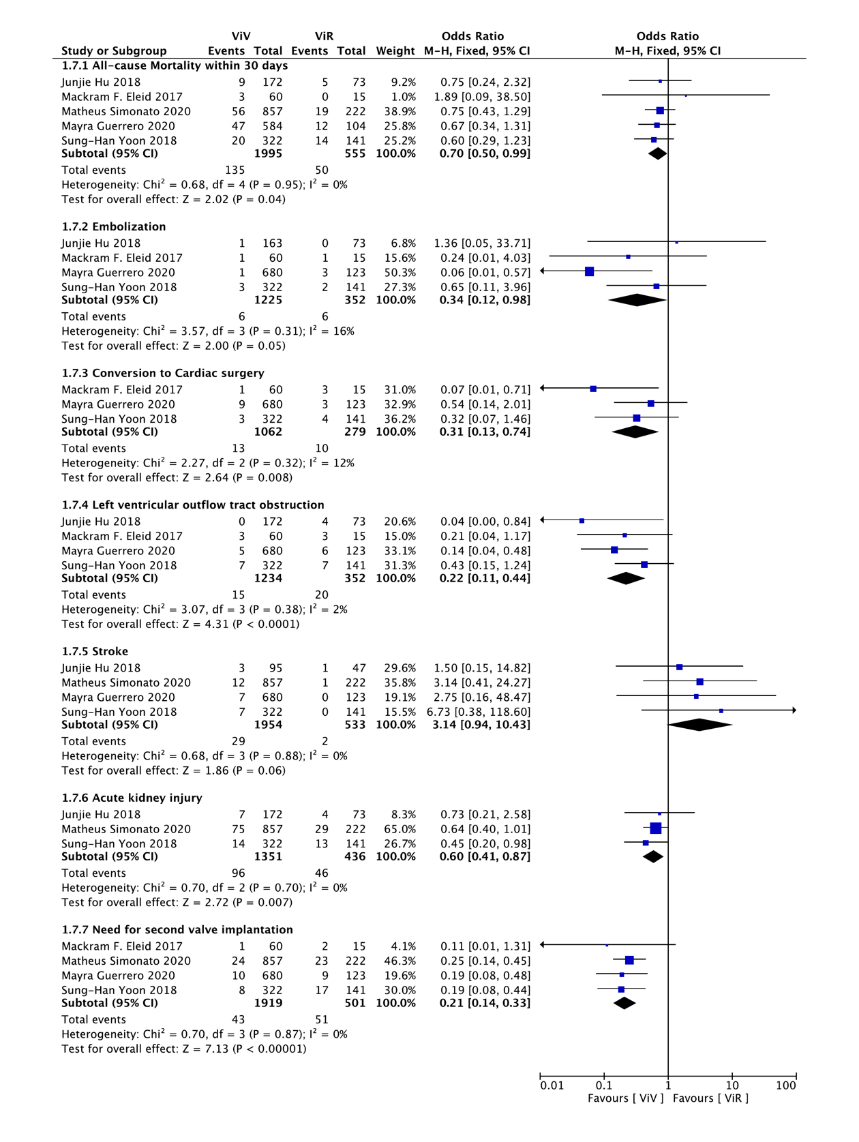


Figure S14 Subgroup of all-cause mortality within 30 days between the group of ViV and ViR. The type of data analyzed was Dichotomous using the Fixed effects.


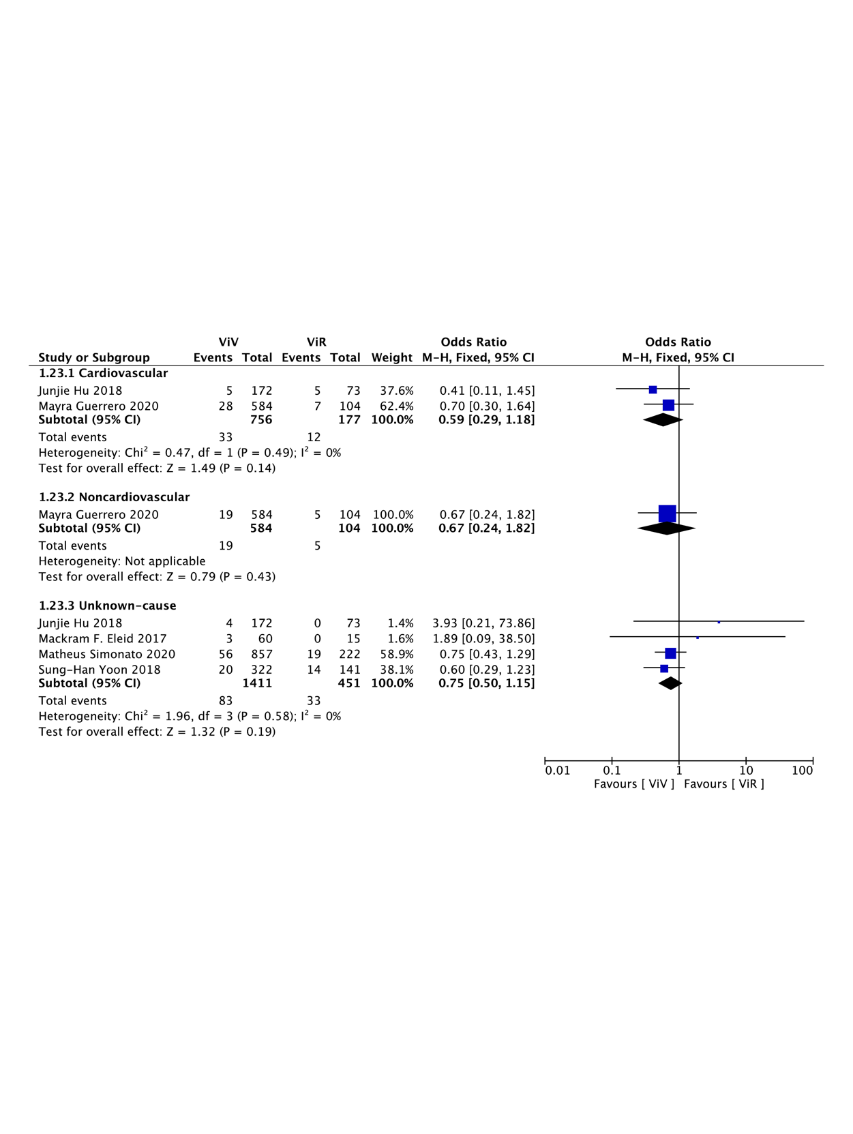


Figure S15 Comparison of Outcomes between the group of ViV and ViR. The type of data analyzed was Continuous (MD) using the Fixed effects.


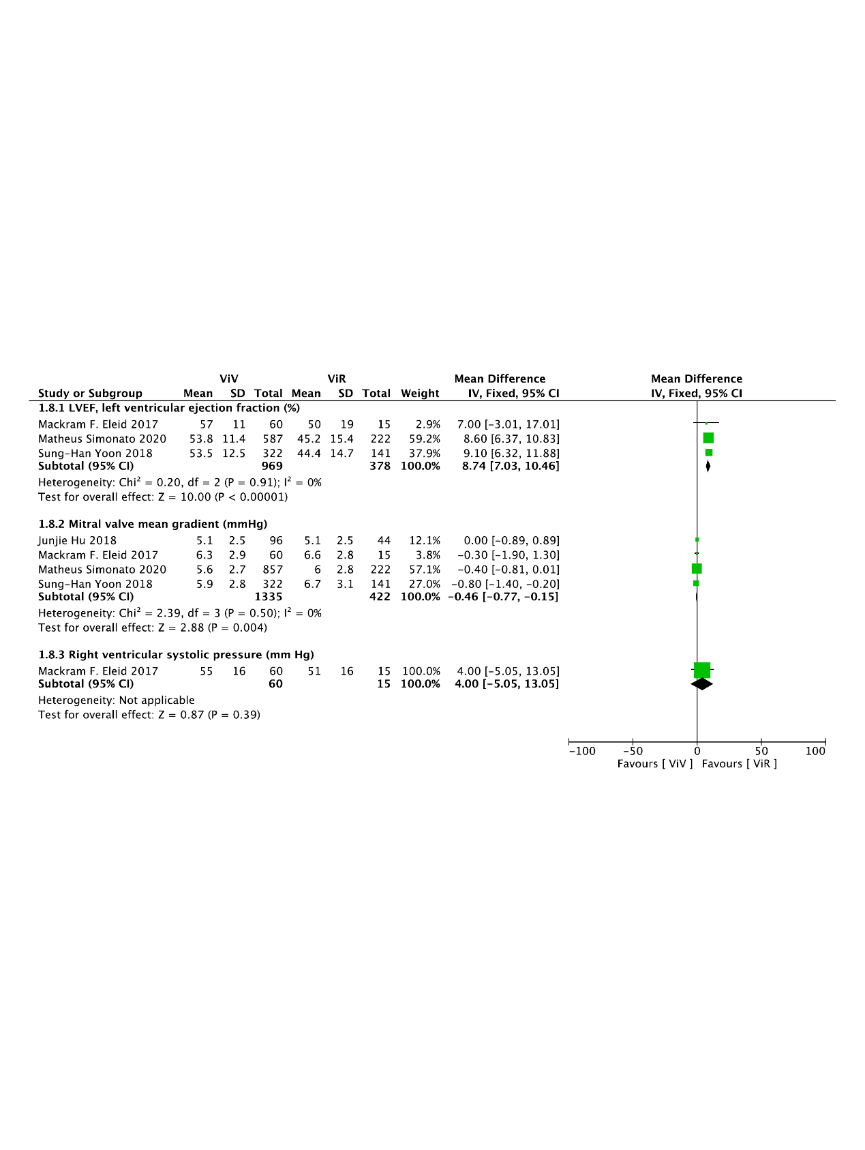


Figure S16 Comparison of Outcomes between the group of ViV and ViR. The type of data analyzed was Continuous (SMD) using the Fixed effects.


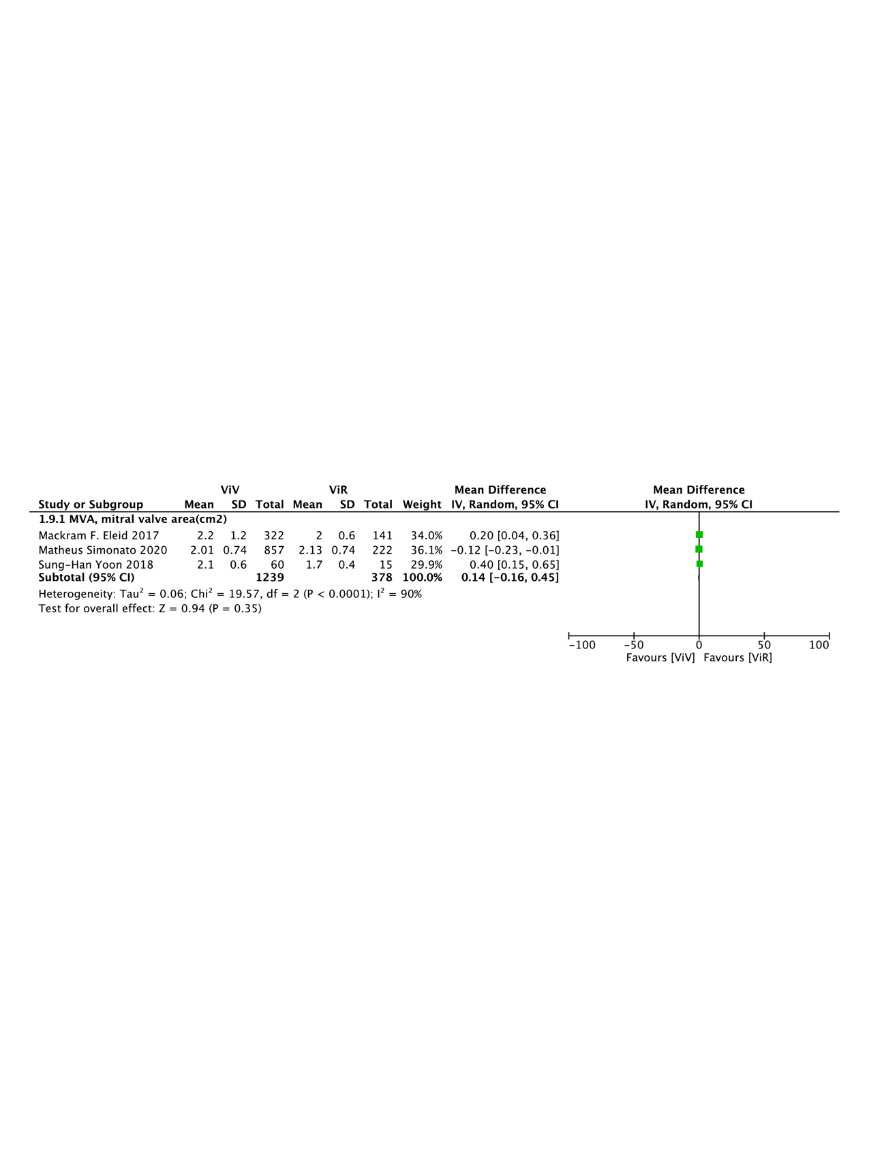


Figure S17 Comparison of Outcomes between the group of ViMAC and ViR. The type of data analyzed was Dichotomous using the Random effects.


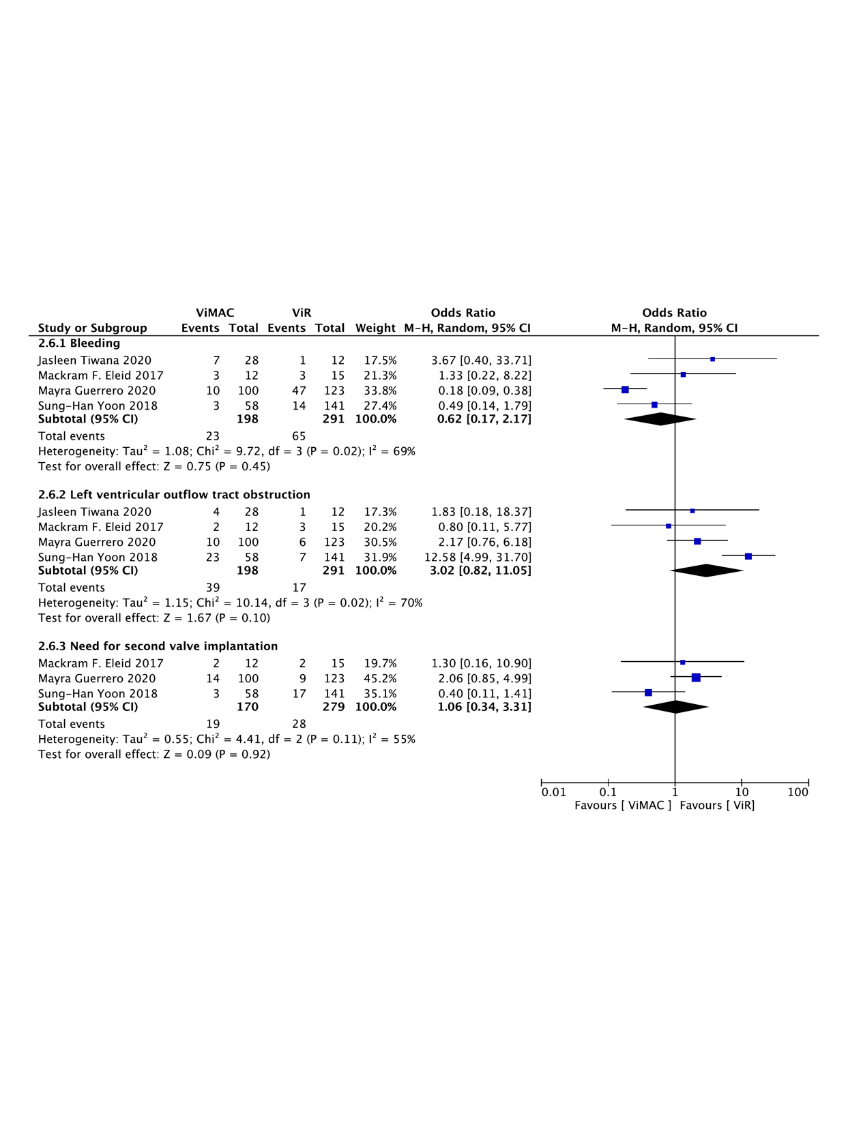


Figure S18 Subgroup of Bleeding between the group of ViMAC and ViR. The type of data analyzed was Dichotomous using the Fixed effects.


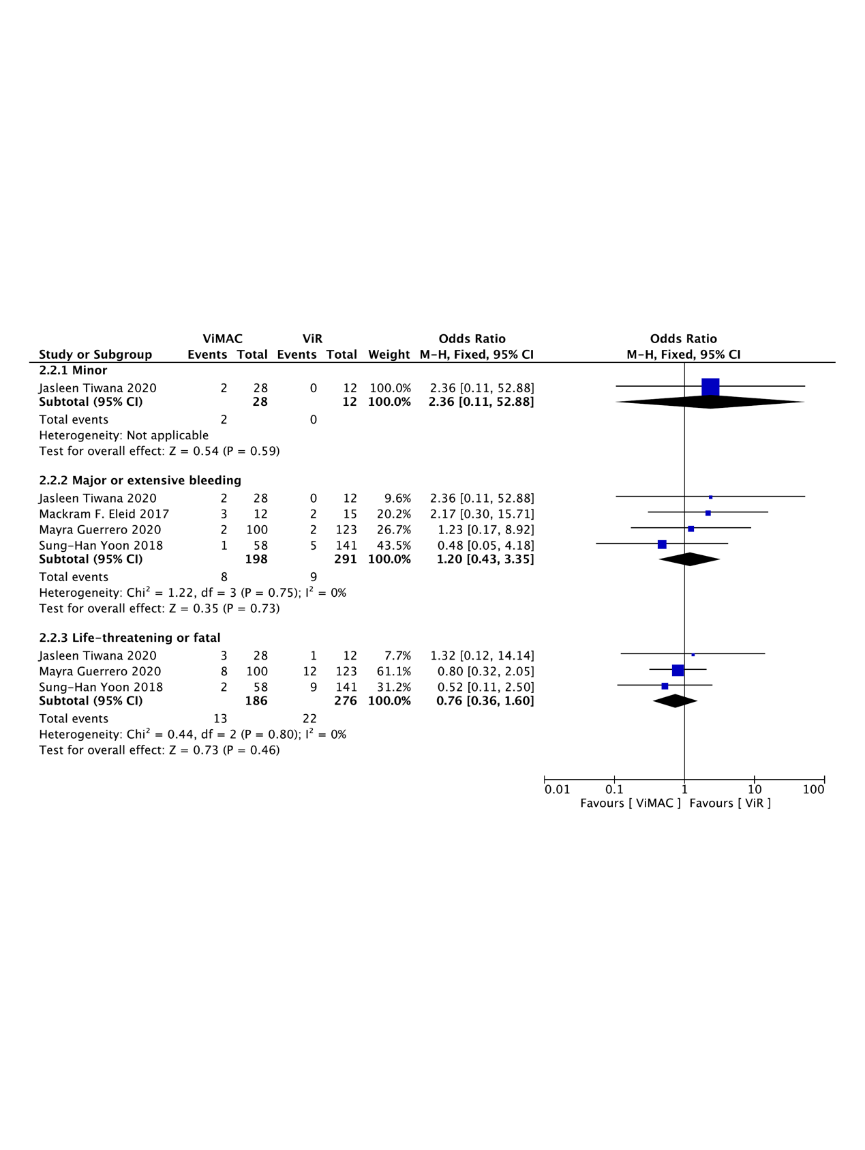


Figure S19 Comparison of Outcomes between the group of ViMAC and ViR. The type of data analyzed was Dichotomous using the Fixed effects.


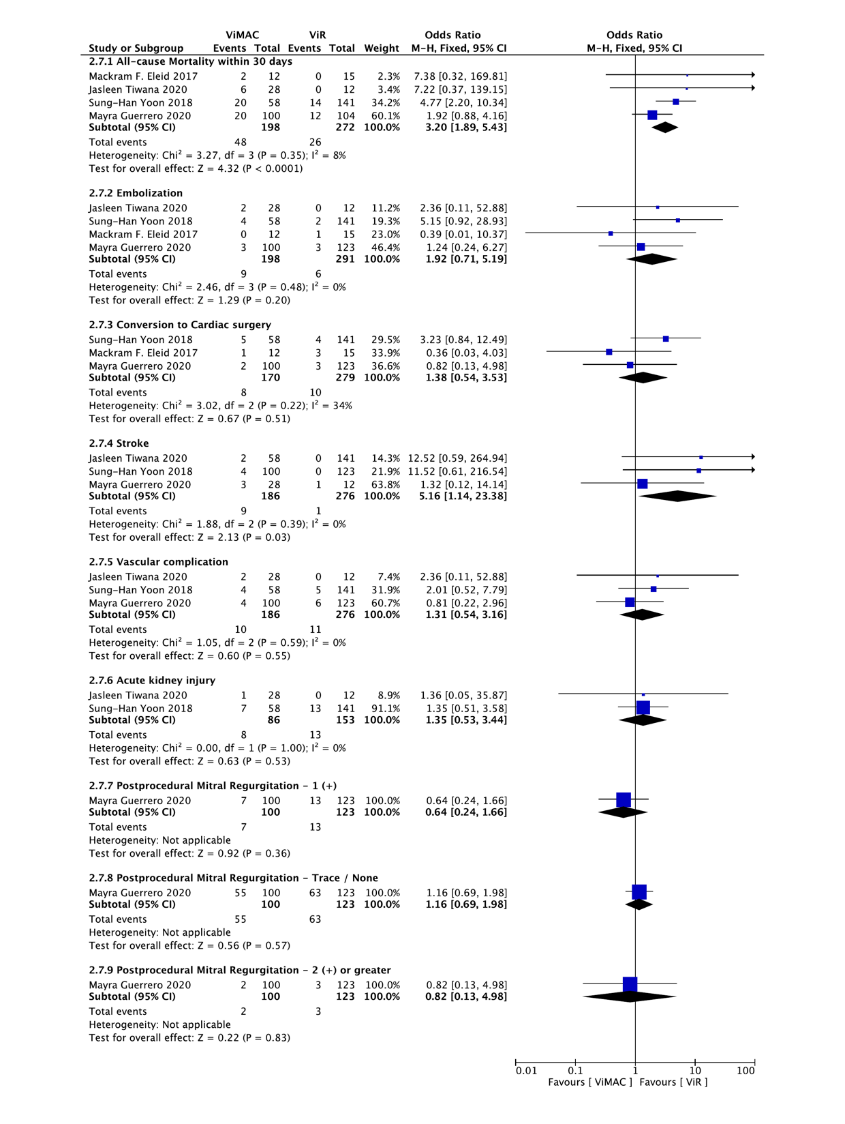


Figure S20 Subgroup of all-cause mortality within 30 days between the group of ViMAC and ViR. The type of data analyzed was Dichotomous using the Fixed effects.


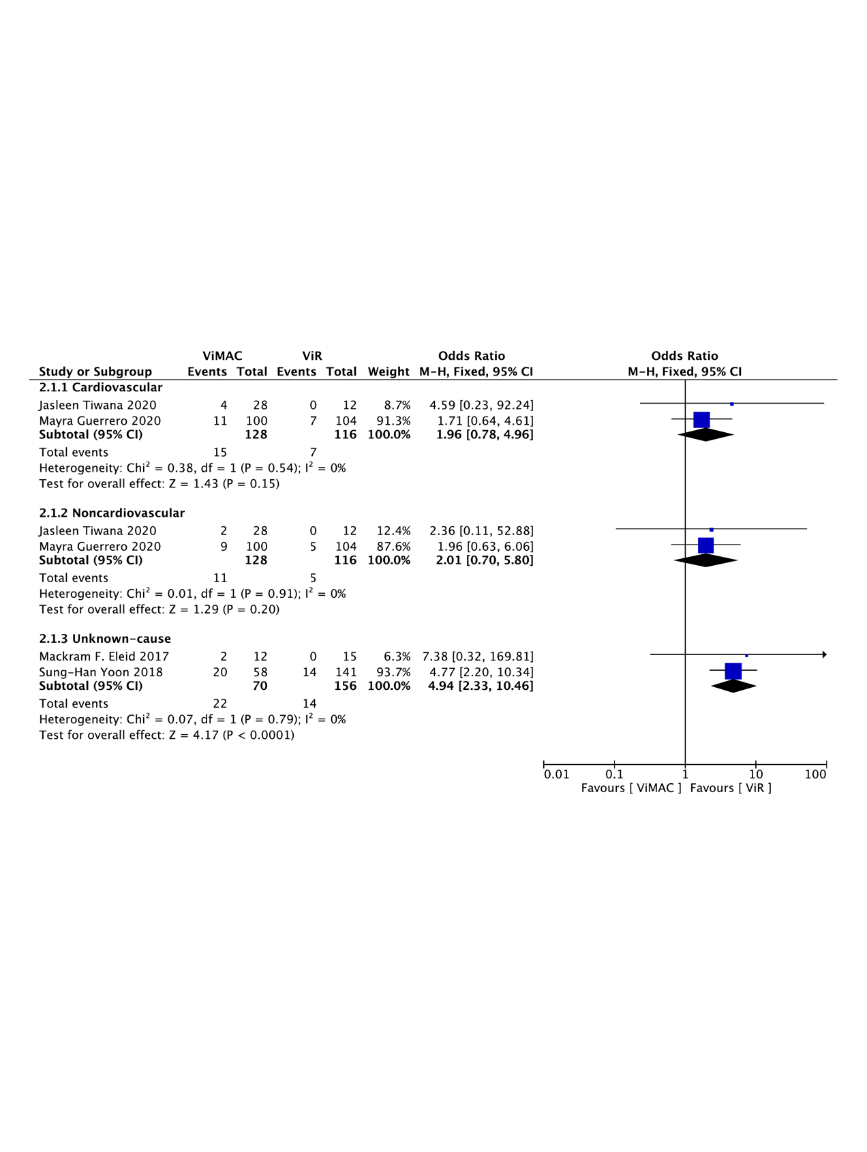


Figure S21 Comparison of Outcomes between the group of ViMAC and ViR. The type of data analyzed was Continuous (MD) using the Fixed effects.


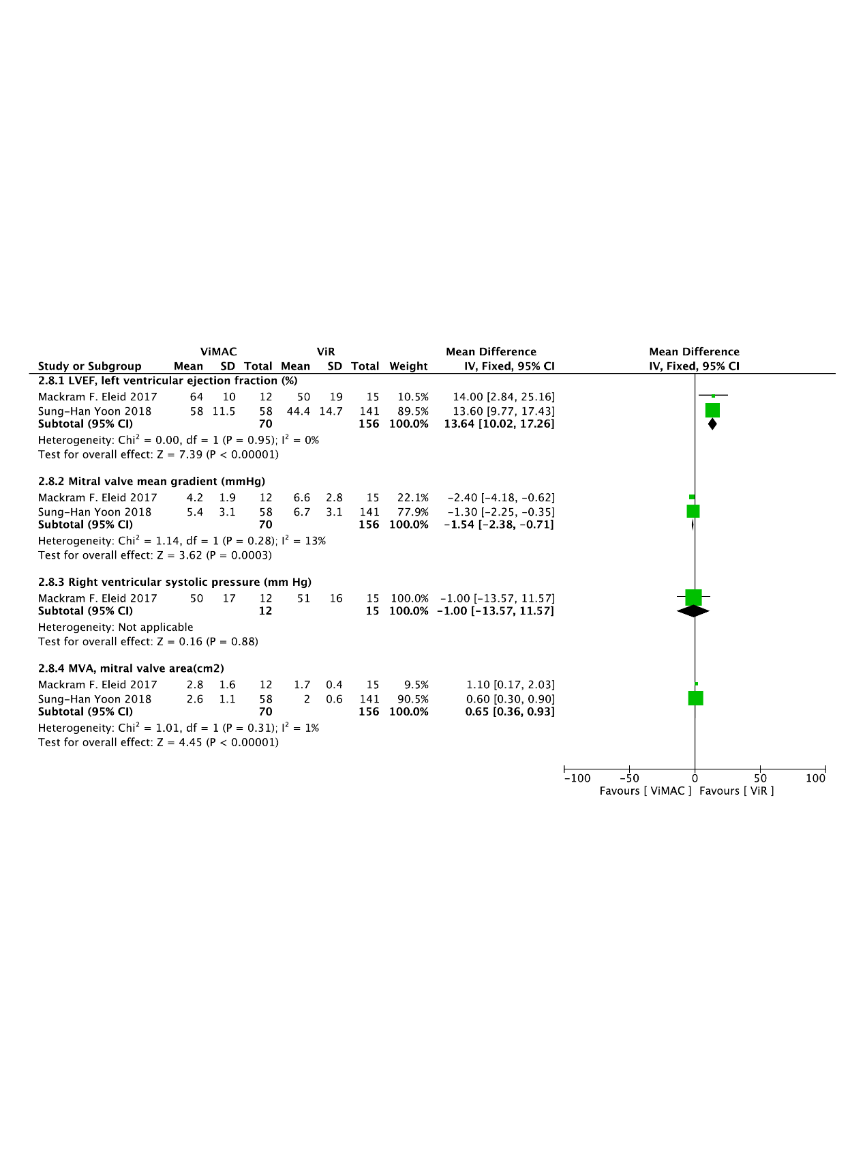

Supplement: Supplementary file 1 — Additional file 1: Fig. S1. Comparison of baseline information between the group of ViV and ViR. The type of data analyzed was Dichotomous using the Random effects. Fig. S2. Comparison of baseline information between the group of ViV and ViR. The type of data analyzed was Dichotomous using the Fixed effects. Fig. S3. Comparison of baseline information between the group of ViV and ViR. The type of data analyzed was Continuous (MD) using the Random effects. Fig. S4. Comparison of baseline information between the group of ViV and ViR. The type of data analyzed was Continuous (MD) using the Fixed effects. Fig. S5. Comparison of baseline information between the group of ViV and ViR. The type of data analyzed was Continuous (SMD) using the Fixed effects. Fig. S6. Comparison of baseline information between the group of ViMAC and ViR. The type of data analyzed was Dichotomous using the Random effects. Fig. S7. Comparison of baseline information between the group of ViMAC and ViR. The type of data analyzed was Dichotomous using the Fixed effects. Fig. S8. Comparison of baseline information between the group of ViMAC and ViR. The type of data analyzed was Continuous (MD) using the Random effects. Fig. S9. Comparison of baseline information between the group of ViMAC and ViR. The type of data analyzed was Continuous (MD) using the Fixed effects. Fig. S10. Comparison of baseline information between the group of ViMAC and ViR. The type of data analyzed was Continuous (SMD) using the Fixed effects. Fig. S11. Comparison of Outcomes between the group of ViV and ViR. The type of data analyzed was Dichotomous using the Random effects. Fig. S12. Subgroup of Bleeding between the group of ViV and ViR. The type of data analyzed was Dichotomous using the Random effects. Fig. S13. Comparison of Outcomes between the group of ViV and ViR. The type of data analyzed was Dichotomous using the Fixed effects. Fig. S14. Subgroup of all-cause mortality within 30 days between the group of ViV and Vi [file 13019_2021_1677_MOESM1_ESM.docx]
